# Genetic diversity of United States Rambouillet, Katahdin and Dorper sheep

**DOI:** 10.1186/s12711-024-00905-7

**Published:** 2024-07-30

**Authors:** Gabrielle M. Becker, Jacob W. Thorne, Joan M. Burke, Ronald M. Lewis, David R. Notter, James L. M. Morgan, Christopher S. Schauer, Whit C. Stewart, R. R. Redden, Brenda M. Murdoch

**Affiliations:** 1 Department of Animal, Veterinary and Food Science, University of Idaho, Moscow, ID USA; 2 Texas A&M AgriLife Extension, Texas A&M University, San Angelo, TX USA; 3 USDA, ARS, Dale Bumpers Small Farms Research Center, Booneville, AR USA; 4 Department of Animal Science, University of Nebraska–Lincoln, Lincoln, NE USA; 5 School of Animal Sciences, Virginia Tech, Blacksburg, VA USA; 6 Round Mountain Consulting, Fayetteville, AR USA; 7 Hettinger Research Extension Center, North Dakota State University, Hettinger, ND USA; 8 Department of Animal Science, University of Wyoming, Laramie, WY USA

## Abstract

**Background:**

Managing genetic diversity is critically important for maintaining species fitness. Excessive homozygosity caused by the loss of genetic diversity can have detrimental effects on the reproduction and production performance of a breed. Analysis of genetic diversity can facilitate the identification of signatures of selection which may contribute to the specific characteristics regarding the health, production and physical appearance of a breed or population. In this study, breeds with well-characterized traits such as fine wool production (Rambouillet, N = 745), parasite resistance (Katahdin, N = 581) and environmental hardiness (Dorper, N = 265) were evaluated for inbreeding, effective population size (*N*_*e*_), runs of homozygosity (ROH) and Wright’s fixation index (F_ST_) outlier approach to identify differential signatures of selection at 36,113 autosomal single nucleotide polymorphisms (SNPs).

**Results:**

Katahdin sheep had the largest current *N*_*e*_ at the most recent generation estimated with both the GONe and NeEstimator software. The most highly conserved ROH Island was identified in Rambouillet with a signature of selection on chromosome 6 containing 202 SNPs called in an ROH in 50 to 94% of the individuals. This region contained the *DCAF16*, *LCORL* and *NCAPG* genes that have been previously reported to be under selection and have biological roles related to milk production and growth traits. The outlier regions identified through the F_ST_ comparisons of Katahdin with Rambouillet and Dorper contained genes with known roles in milk production and mastitis resistance or susceptibility, and the F_ST_ comparisons of Rambouillet with Katahdin and Dorper identified genes related to wool growth, suggesting these traits have been under natural or artificial selection pressure in these populations. Genes involved in the cytokine-cytokine receptor interaction pathways were identified in all F_ST_ breed comparisons, which indicates the presence of allelic diversity between these breeds in genomic regions controlling cytokine signaling mechanisms.

**Conclusions:**

In this paper, we describe signatures of selection within diverse and economically important U.S. sheep breeds. The genes contained within these signatures are proposed for further study to understand their relevance to biological traits and improve understanding of breed diversity.

**Supplementary Information:**

The online version contains supplementary material available at 10.1186/s12711-024-00905-7.

## Background

Genetic diversity is an important resource in animal production and conservation. Loss of genetic diversity can impact a species or a breed’s ability to adapt to changing environmental or production pressures [1] and can lead to the accumulation of lethal or deleterious alleles with detrimental effects on health and production [2]. Adaptability of animal production requires the preservation of genetic diversity, and the identification of signatures of selection can provide essential insights that can be useful for conservation and breed improvement objectives [3, 4].

Artificial and natural selection both result in changes in allele frequencies that can lead to the fixation of certain alleles [5]. The neutral regions that surround alleles under selection tend to lose genetic variation due to the hitchhiking effect, which increases linkage disequilibrium (LD) and can result in the formation of selective sweeps [6–9]. Analyses of runs of homozygosity (ROH) can be used to detect regions that have experienced loss of heterozygosity due to the presence of selective pressures [10]. Analysis based on Wright’s fixation index (F_ST_) identifies differences in allele frequencies between populations and is one of the most commonly used methods to identify single nucleotide polymorphisms (SNPs) under selection [11–13]. In sheep, these analyses have been used to understand the genomic regions under selection for traits such as environmental adaption [14–17], parasite resistance and susceptibility [18], morphological traits [19–21], wool quality [22], and production traits [23] in many breeds.

The purpose of this study was to evaluate measures of genetic diversity and signatures of selection in three popular U.S. sheep breeds (Rambouillet, Katahdin, and Dorper) with diverse characteristics. The U.S. Rambouillet was first established in 1840 with the import of fine-wool sheep from France [24]; today, Rambouillet is a multi-purpose breed that is noted for wool and carcass quality. Compared to the other breeds of this study, Rambouillet is a larger framed, later-maturing breed [25] and is prominent in the semi-arid western states where much of the U.S. sheep production is concentrated [26]. The Katahdin is a composite hair breed that was developed in the 1950s from St. Croix hair sheep and wool breeds including the Suffolk and Wiltshire Horn [27, 28]. Katahdin is a fast-growing and prolific breed that is regarded for parasite resistance and suitability to warm, tropical environments [29, 30]. Dorper sheep are early-maturing and produce heavily muscled carcasses with many favorable palatability and tenderness characteristics [31]. To develop the Dorper breed, Dorset Horn and fat-rumped Blackhead Persian breeds were crossed to combine maternal traits with wool-shedding ability and adaptability to harsh environmental conditions [32].

Genotype data of Rambouillet, Katahdin, and Dorper animals were analyzed for inbreeding, effective population size (*N*_*e*_), and signatures of selection (through F_ST_ and ROH) in order to gain understanding of the selection pressures that affect these breeds. Genes present in regions under selection were further evaluated to identify the biological pathways that are most likely affected. The results of this study provide insights into the genetic variation present in breeds commonly raised in the U.S. for their meat and wool quality (Rambouillet), parasite resistance (Katahdin), and environmental hardiness (Dorper).

## Methods

### Animal sampling and DNA genotyping

Sheep belonging to the Rambouillet, Katahdin, and Dorper breeds were selected for analyses of inbreeding and genetic diversity. These breeds were chosen to facilitate analyses of sheep that have been selected for diverse purposes, including fine wool (Rambouillet), parasite resistance (Katahdin), and environmental hardiness (Dorper). In total, 745 Rambouillet sheep were sampled from the Texas A&M AgriLife Research flock (TAMU; N = 403) and from central performance ram tests held at the North Dakota State University (NDSU; N = 159) and University of Wyoming (UWY; 183), representing more than 30 seedstock producers located in Colorado, Montana, North Dakota, South Dakota, and Wyoming. The Katahdin sheep (N = 581) were sampled from 20 flocks located across the U.S. (Arkansas, Georgia, Idaho, Indiana, Missouri, New York, Ohio, Oregon, Texas, Virgina, West Virginia, and Wisconsin). The Dorper sheep were sampled from the TAMU research flock (N = 265), which was founded from Dorper or White Dorper rams and ewes incorporated from over 20 producers throughout the U.S. in the early 2000s [33]. Over the last two decades, this flock has been managed as one cohort. In total, 1591 sheep were analyzed in the current study.

Sample collection and genotyping of these sheep have been described previously [34–36]. Briefly, DNA extraction and genotyping of Katahdin sheep were conducted at Neogen Corporation-GeneSeek Operations, Lincoln, NE, USA, whereas DNA from the Rambouillet and Dorper animals was extracted from whole blood samples using the phenol–chloroform method or from tissue samples by AgResearch (GenomNZ, AgResearch, New Zealand) [37]. Genotyping was conducted with the high-density (HD) Illumina 600K SNP BeadChip (Illumina Inc., San Diego, CA, USA) that includes 606,006 SNPs, the Applied Biosystems™ Axiom™ Ovine Genotyping Array (50K) that includes 51,572 SNPs (Thermo Fisher Scientific, catalog number 550898), or the AgResearch Sheep Genomics 60K SNP chip that includes 68,848 SNPs (GenomNZ, AgResearch, New Zealand). In total, 132 Dorper and 243 Rambouillet samples were genotyped on the AgResearch chip, 133 Dorper and 502 Rambouillet samples were genotyped on the Applied Biosystems array, and the 581 Katahdin samples were genotyped on the HD Illumina chip.

Duplicate markers within a panel were filtered to retain the SNPs with the highest call rate (CR) at each unique position. Compatible SNPs across panels were merged using the Plink v1.9 software [38, 39]. Non-autosomal SNPs and SNPs with a CR lower than 90% were removed, which resulted in a dataset of 36,113 SNPs. The 1591 sheep had a genotype CR ≥ 90%. Removing SNPs with a low minor allele frequency (MAF) or that are in high LD can hinder the detection of ROH [40] and MAF thresholds can affect the comparison of structure between populations [41]. For these reasons, no further filtering was applied to the data prior to principal component analysis (PCA), ROH analyses, Wright’s fixation index (F_ST_) outlier approach, and *N*_*e*_ estimation.

The Rambouillet and Katahdin sheep analyzed in this study were sampled from geographically distant flocks and can be considered representative of their breed in the context of the U.S. sheep industry. Due to a limited representation of the current breed diversity in the sampled Dorper animals, the allele frequencies reported in these sheep are not anticipated to be representative of the U.S. Thus, for the Dorper breed, although analyses of these animals are still valuable and worth reporting, care should be taken to avoid over interpretation. For this reason, the Dorper ROH and *N*_*e*_ results are shared as supplementary data. The Dorper data were used in the pairwise F_ST_ signatures of selection analyses to facilitate interpretation of outlier SNPs that are potentially under selection in the Rambouillet and Katahdin breeds.

### Population structure, inbreeding, and effective population size

The population structure between breeds was visualized through PCA that was conducted with the Plink v1.9 software. The results were visualized with the package ggplot2 in R version 4.2.3, with the first principal component (PC1) plotted on the x-axis and PC2 plotted on the y-axis [42–44]. The proportions of variance explained (PVE) by PC1 and PC2 were calculated by dividing the first and second eigenvalues, respectively, by the sum of all eigenvalues.

To understand the level of inbreeding within each breed, homozygosity was evaluated through the proportion of observed and expected homozygous SNPs and through two methods of inbreeding calculations. The number of expected and observed homozygous SNPs and the method-of-moments inbreeding coefficient (F) were calculated using Plink v1.9 (–het) for each animal [45]. The F statistic is calculated as ([observed homozygous count] − [expected count])/([total observations] − [expected count]). ROH-based inbreeding coefficients were calculated with the package detectRuns using the parameters described for the ROH analysis. The genome-wide ROH-based inbreeding coefficient, F_ROH_, was calculated as the sum of the individual’s ROH length over the length of the genome [46]. The distribution of F_ROH_ by breed was visualized with ggplot2. The Kruskal–Wallis test was used as a non-parametric alternative to the one-way analysis of variance (ANOVA) to determine whether there were statistical differences in F or F_ROH_ between breeds [47]. The Kruskal–Wallis test was followed by the Dunn’s test to calculate *P-*values for pairwise breed comparisons [48].

The *N*_*e*_ of each breed was estimated using three methods. Historic *N*_*e*_ was estimated using the LD-based method in the SNeP software version 1.11 for each breed [49]. Default parameters were set for each analysis. LD was measured using *r*^*2*^, the squared correlation coefficient between pairs of SNPs [50]. The rate of the decline in *N*_*e*_ was calculated between each consecutive reported generation and for the overall distribution. The NeEstimator v2.1 software was used to estimate current *N*_*e*_ for each breed using the LD method within the random mating model [51]. For comparison, current and historic *N*_*e*_ were also estimated using the GONe software [52], with default parameters. Results of SNeP, NeEstimator, and GONe were visualized in R with ggplot2.

### Runs of homozygosity and F_ST_ analyses

Analysis of ROH was conducted to identify regions which may be under selection pressure in the Rambouillet, Katahdin, and Dorper breeds. The ROH were identified using the sliding window method with the R package detectRuns [46]. Each window comprised 15 SNPs with a maximum of two opposite SNP genotypes (heterozygous/homozygous) and one missing SNP allowed per window. A ROH was required to have a minimum length of 250,000 bp and to contain at least 30 SNPs. There was a minimum density of one SNP per every 10,000 kb and a maximum gap between SNPs of 10,000 kb. A homozygous window threshold of 0.05 was used to determine SNP inclusion in a ROH. Results were visualized using the CMPlot, ggplot2, and patchwork packages in R [53, 54]. The regions that contained a ROH in more than 50% of the individuals of a breed were selected for further analyses.

The average length of ROH called in Katahdin and Rambouillet were investigated for significant differences between breeds. All data were evaluated for normality with the Shapiro-Wilks test prior to model selection with the ‘shapiro.test’ function in R [44, 55]. The Wilcoxon unpaired two-sample test was performed using the ‘compare_means’ function in the ggpubr package of R [43, 56].

To better understand the genetic differences between breeds, Wright’s fixation index (F_ST_) was calculated in Plink v2.0 using the method proposed by Weir and Cockerham [39, 57]. Analysis with Plink v2.0 was preferred over Plink v1.9 because of its ability to generate pairwise F_ST_ output files simultaneously for all comparisons. Per-variant F_ST_ estimates were generated for each SNP and genome-wide F_ST_ estimates were reported as ratio-of-averages between each pair of breeds, which were calculated from the ratio of the average variance components [58]. Fisher’s exact test in R was used to estimate p-values from contingency tables constructed with genotype counts for each SNP and each pair of breeds. Outlier SNPs with F_ST_ estimates greater than three standard deviations above the mean and with significant Fisher’s test *P*-values (< 0.05) were considered to differ greatly between breeds. For each pairwise breed analysis, F_ST_ regions of interest were defined by significant outlier SNPs located within 200,000 bp of each other, ± 100,000 bp before and after the flanking markers. For the purpose of comparison, F_ST_ was also estimated through the BayeScan 2.1 software with default parameters [59]. Genotype files were prepared for import into BayesScan using the ‘genomic_converter’ tool in the R package radiator [60].

The identified ROH and F_ST_ regions were queried through the National Center for Biotechnology Information (NCBI) genome browser tool [61, 62]. The ARS UI_Ramb_v2.0 genome assembly [63] was used for all SNP positions and gene region analyses. Characterized genes falling within a ROH or F_ST_ region were recorded for pathway enrichment analyses. Where possible, the predicted human ortholog of *Ovis aries* genes containing the LOC symbol (pseudo or uncharacterized genes) were recorded.

### Pathway analyses

Genes of interest from ROH and F_ST_ analyses were investigated to identify associated biological pathways. Pathway enrichment analyses were conducted through the Gene Ontology (GO) and the Kyoto Encyclopedia of Genes and Genomes (KEGG) Mapper search tools [64–67]. Due to reference database availability, queries through GO for biological process, molecular function, and cellular component were made against the *Bos taurus* reference database and queries of KEGG Mapper were made against the *Homo sapiens* reference. Pathway analyses were conducted for genes located within Rambouillet, Katahdin, or Dorper ROH islands and for genes located within Katahdin-Rambouillet, Katahdin-Dorper, or Rambouillet-Dorper F_ST_ regions. In addition, pathway analyses were conducted for genes which were in common between both F_ST_ analyses of Rambouillet (Katahdin-Rambouillet and Rambouillet-Dorper) or both F_ST_ analyses of Katahdin (Katahdin-Rambouillet and Katahdin-Dorper). To better understand the biological implications of genes within ROH islands and F_ST_ outlier regions, the STRING database was queried for protein–protein interaction networks and functional enrichment analysis of these genes [68].

## Results

### Population structure, inbreeding, and effective population size

The population structure of the studied animals was first evaluated through PCA. The analysis revealed three clearly distinct clusters, with Rambouillet, Katahdin, and Dorper sheep clustering more closely with individuals of the same breed than individuals of other breeds (see Additional file 1: Fig. S1). The model’s PC1 had an eigenvalue of 179.82 and was estimated to explain 35.99% of all the variation while PC2 had an eigenvalue of 116.82 and was estimated to explain 23.38% of the variation. The largest principal component separated the Katahdin and Rambouillet breeds, while PC2 separated Dorper from both Katahdin and Rambouillet. The placement of these breeds is similar to the results of previous PCA, which have shown the separation of breeds originating from West Africa, South Africa, and the Iberian Peninsula [69, 70].

**Fig. 1:**
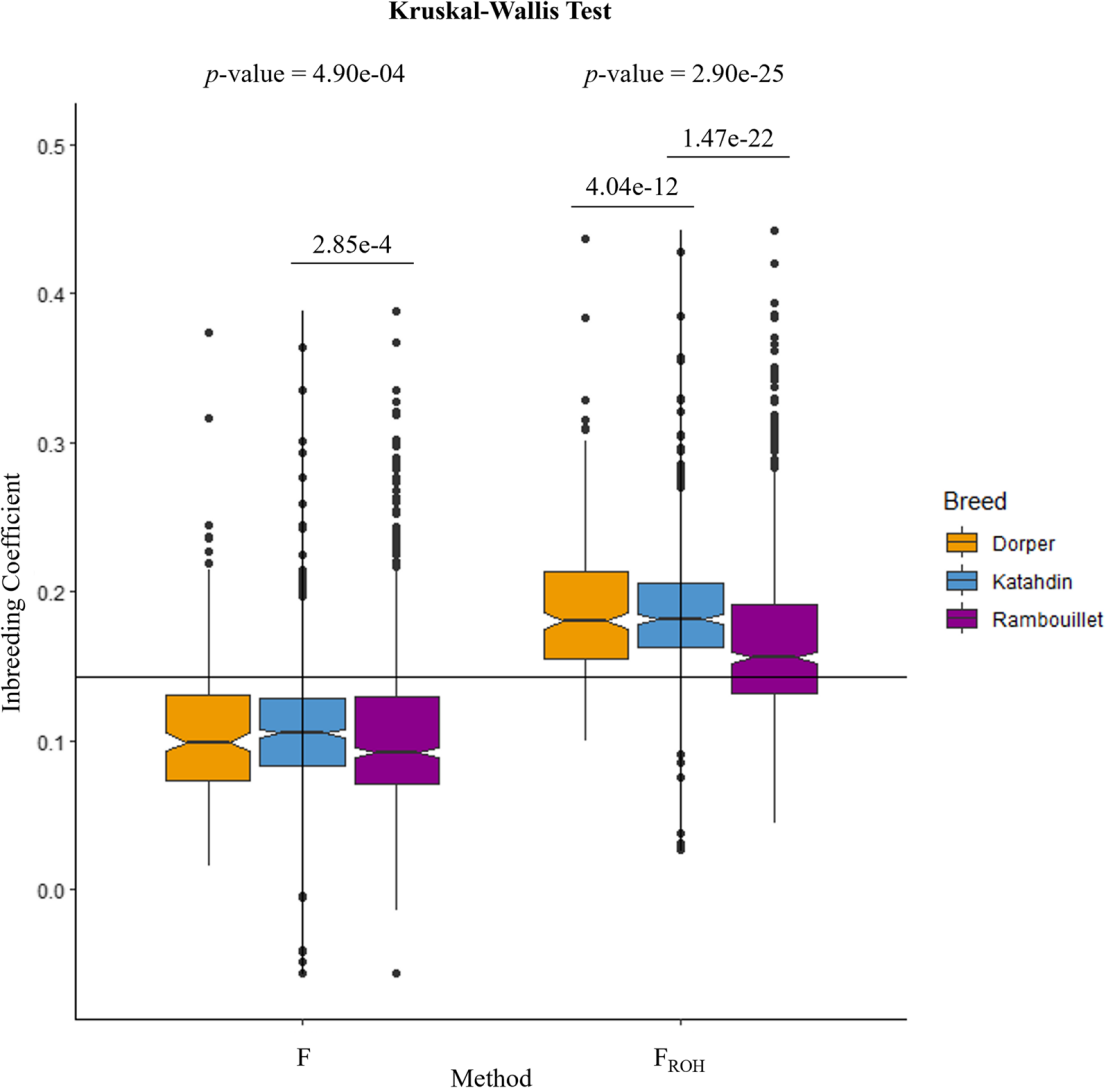
Distribution of F and F_ROH_ inbreeding coefficients by breed. Boxplots of the distribution of F and F_ROH_ for Dorper, Katahdin, and Rambouillet breeds. The horizontal black line represents the overall mean inbreeding coefficient calculated from both F and F_ROH_ estimates

The average proportion of expected and observed homozygous SNPs for Rambouillet, Katahdin, and Dorper are reported in Table 1. The observed homozygosity was similar to previous estimates for Rambouillet [71] and was slightly higher than previous estimates for Katahdin and Dorper [72, 73]. For each breed, the average F_ROH_ estimate was higher than the average F estimate. The Kruskal–Wallis *P*-value for ‘F ~ Breed’ was 4.90e−04, and for ‘F_ROH_ ~ Breed’ was 2.89e−25. Based on the Dunn’s test, Katahdin had significantly higher F inbreeding coefficients than Rambouillet; for F_ROH_, the Rambouillet breed had significantly lower inbreeding coefficients than both Dorper and Katahdin (Fig. 1). Overall, F_ROH_ estimates were higher than previously reported estimates for worldwide sheep populations [74, 75]. Previous studies have sampled fewer individuals per breed, which may contribute to these observations.

**Table 1:** Homozygosity and inbreeding coefficients for the Dorper, Katahdin, and Rambouillet breeds

Breed	Expected Hom. AVG ± SD	Observed Hom. AVG ± SD	F AVG ± SD	Range F	F_ROH_ AVG ± SD	Range F_ROH_
Rambouillet	0.6216 ± 0.0002	0.6620 ± 0.0218	0.1069 ± 0.06	− 0.057 to 0.389	0.1690 ± 0.06	0.044–0.442
Katahdin	0.6216 ± 0.0002	0.6628 ± 0.0169	0.1090 ± 0.04	− 0.057 to 0.364	0.1865 ± 0.04	0.027–0.428
Dorper	0.6215 ± 0.0001	0.6619 ± 0.0182	0.1067 ± 0.05	0.016 to 0.374	0.1875 ± 0.05	0.099–0.437

F is the method-of-moments inbreeding coefficient, F_ROH_ is the ROH-based inbreeding coefficient

Hom.: homozygosity; SD: standard deviation

**Fig. 2:**
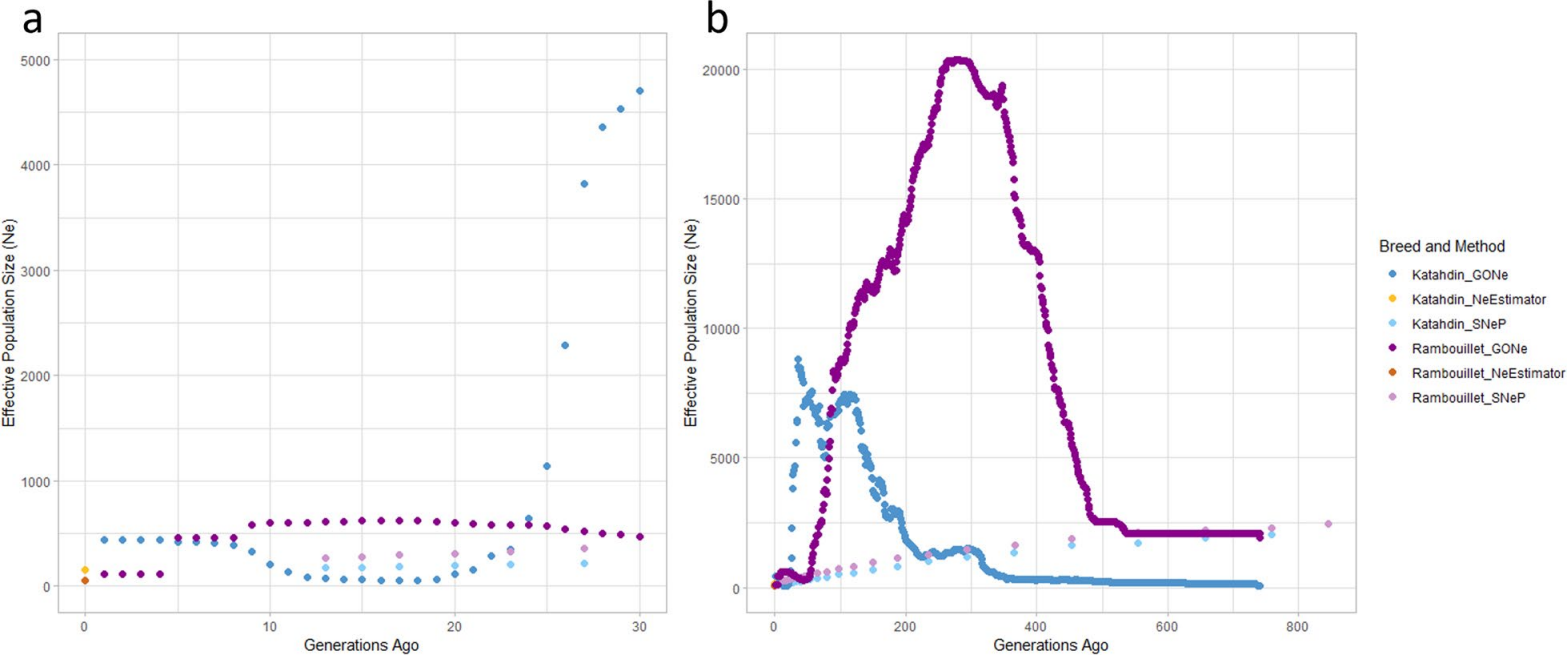
Recent and historic *N*_*e*_ estimated through three methods. **a** *N*_*e*_ estimates from 30 generations ago to the current one for Katahdin and Rambouillet, estimated with GONe, NeEstimator, and SNeP; **b** Historic *N*_*e*_ estimates for Katahdin and Rambouillet estimated with GONe and SNeP

Estimates of *N*_*e*_ were calculated using the SNeP, NeEstimator, and GONe software. The *N*_*e*_ of the Rambouillet and Katahdin breeds were estimated from 759 generations ago to 13 generations ago using SNeP, from 741 generations ago to one generation ago using GONe, and for the current generation (given the notation 0) with NeEstimator (Fig. 2). Estimations with GONe were nonlinear, while estimations with SNeP showed a consistent decline in *N*_*e*_ from the furthest generation to the most recent. These general observations of SNeP and GONe trajectories are similar to the *N*_*e*_ results reported in the Master thesis work of D Adepoju with cattle data [76]. The GONe and SNeP estimates began to show more agreement approximately 30 generations ago (Fig. 2a). In the most recent generation, Katahdin were estimated to have an *N*_*e*_ of 161.4 by NeEstimator or 436.1 by GONe, while Rambouillet had *N*_*e*_ estimates of 56.9 by NeEstimator or 111.8 by GONe (Fig. 2b).

The rate of change (m) of *N*_*e*_ size over generational time can give insight into the rate of diversity loss. These calculations were made with the results of SNeP, as these estimates followed a linear pattern. The overall rate of change was m = 3.02 for Rambouillet and m = 2.76 for Katahdin, suggesting that the Rambouillet breed has lost genetic diversity at a slightly more rapid rate than the Katahdin breed. The most rapid decrease in *N*_*e*_ occurred between 80 and 98 generations ago for the Katahdin (m = 5.11; Table 2) and between 15 and 17 generations ago for the Rambouillet breed (m = 9.00; Table 3). Katahdin also had a high rate of change 15 to 17 generations ago (m = 5, Table 3). In addition, both Rambouillet and Katahdin showed higher rates of change between 20 and 23 generations ago, with Rambouillet having an m of 7.33, and Katahdin having an m of 5.00 between these intervals. Each subsequent generation had a lower *N*_*e*_ estimate than the preceding generation, and in each generation the estimate for Rambouillet was greater than that for Katahdin. The *N*_*e*_ estimates for these breeds became more similar as the generations became closer to the current one. Analysis results for the Dorper sheep are reported as supplementary data (see Additional file 2: Table S1). The greatest rate of change for Dorper was estimated from 17 to 20 generations ago, with m = 4.00, and the overall rate of change was m = 2.05.

**Table 2:** Effective population size (*N*_*e*_) for Katahdin sheep

Generations ago	*N*_*e*_	Average distance (bp)	Average LD (${{\varvec{r}}}^{2}$) ± SD	Number of pairwise comparisons	Rate of change (m)
13	178	3,749,388	0.0361 ± 0.05	38,212	0.50
15	179	3,272,881	0.0410 ± 0.06	34,640	5.00*
17	189	2,843,420	0.0445 ± 0.07	31,503	1.67
20	194	2,460,514	0.0497 ± 0.07	28,092	5.00*
23	209	2,116,735	0.0535 ± 0.08	25,271	2.00
27	217	1,811,459	0.0597 ± 0.08	22,366	4.40
32	239	1,541,337	0.0637 ± 0.09	19,760	2.67
38	255	1,303,408	0.0700 ± 0.10	17,620	4.14
45	284	1,095,495	0.0745 ± 0.10	15,484	3.67
54	317	914,182	0.0795 ± 0.11	13,424	4.55
65	367	757,894	0.0825 ± 0.11	11,640	3.40
80	418	623,867	0.0876 ± 0.12	10,128	5.11*
98	510	509,861	0.0878 ± 0.12	8529	3.27
120	582	413,447	0.0941 ± 0.13	7034	4.07
150	704	333,299	0.0962 ± 0.13	5838	2.59
187	800	266,817	0.1049 ± 0.14	4792	4.43
234	1008	213,215	0.1042 ± 0.14	3832	3.20
293	1197	170,203	0.1093 ± 0.14	3189	2.03
366	1345	136,424	0.1199 ± 0.16	2460	3.24
454	1630	110,075	0.1223 ± 0.16	1872	1.00
554	1730	90,236	0.1380 ± 0.18	1428	1.82
657	1917	76,085	0.1464 ± 0.19	939	1.45
759	2065	65,804	0.1554 ± 0.20	698	*Overall 2.76*

The rate of change in *N*_*e*_ (m) was calculated as the change in *N*_*e*_ over the change in generations ago (∆*N*_*e*_/∆generation). The greatest rates of change are indicated with (*) and the overall rate of change is given in italics

**Table 3:** Effective population size *(N*_*e*_*)* for Rambouillet sheep

Generations ago	*N*_*e*_	Average distance (bp)	Average LD (${{\varvec{r}}}^{2}$) ± SD	Number of pairwise comparisons	Rate of change (m)
13	268	3,748,453	0.0243 ± 0.04	39,003	4.00
15	276	3,272,995	0.0269 ± 0.04	35,589	9.00*
17	294	2,843,334	0.0291 ± 0.04	31,860	5.67
20	311	2,459,704	0.0316 ± 0.05	28,845	7.33*
23	333	2,116,548	0.0343 ± 0.05	25,565	6.50*
27	359	1,810,664	0.0370 ± 0.05	23,218	5.00
32	384	1,541,490	0.0406 ± 0.06	20,609	5.83
38	419	1,303,677	0.0437 ± 0.06	18,002	4.86
45	453	1,095,627	0.0479 ± 0.07	15,746	6.00
54	507	914,251	0.0512 ± 0.07	13,836	5.45
65	567	757,660	0.0550 ± 0.08	11,965	3.20
80	615	623,577	0.0612 ± 0.09	10,246	5.89
98	721	509,735	0.0637 ± 0.09	8553	3.39
121	799	413,170	0.0704 ± 0.10	7279	6.68*
149	986	333,486	0.0707 ± 0.10	5903	4.08
187	1141	267,022	0.0758 ± 0.10	4976	2.62
234	1264	213,570	0.0848 ± 0.12	4033	3.80
293	1488	170,488	0.0897 ± 0.12	3228	2.27
367	1656	136,133	0.0998 ± 0.14	2507	2.63
454	1885	110,105	0.1075 ± 0.14	1972	2.65
554	2150	90,160	0.1143 ± 0.16	1415	0.63
657	2215	75,994	0.1293 ± 0.17	959	0.69
759	2285	65,817	0.1425 ± 0.18	702	*Overall 3.02*

The rate of change in *N*_*e*_ (m) was calculated as the change in *N*_*e*_ over the change in generations ago (∆*N*_*e*_/∆generation). The greatest rates of change are indicated with (*) and the overall rate of change is given in italics

### Runs of homozygosity and Wright’s F_ST_ analyses

**Fig. 3:**
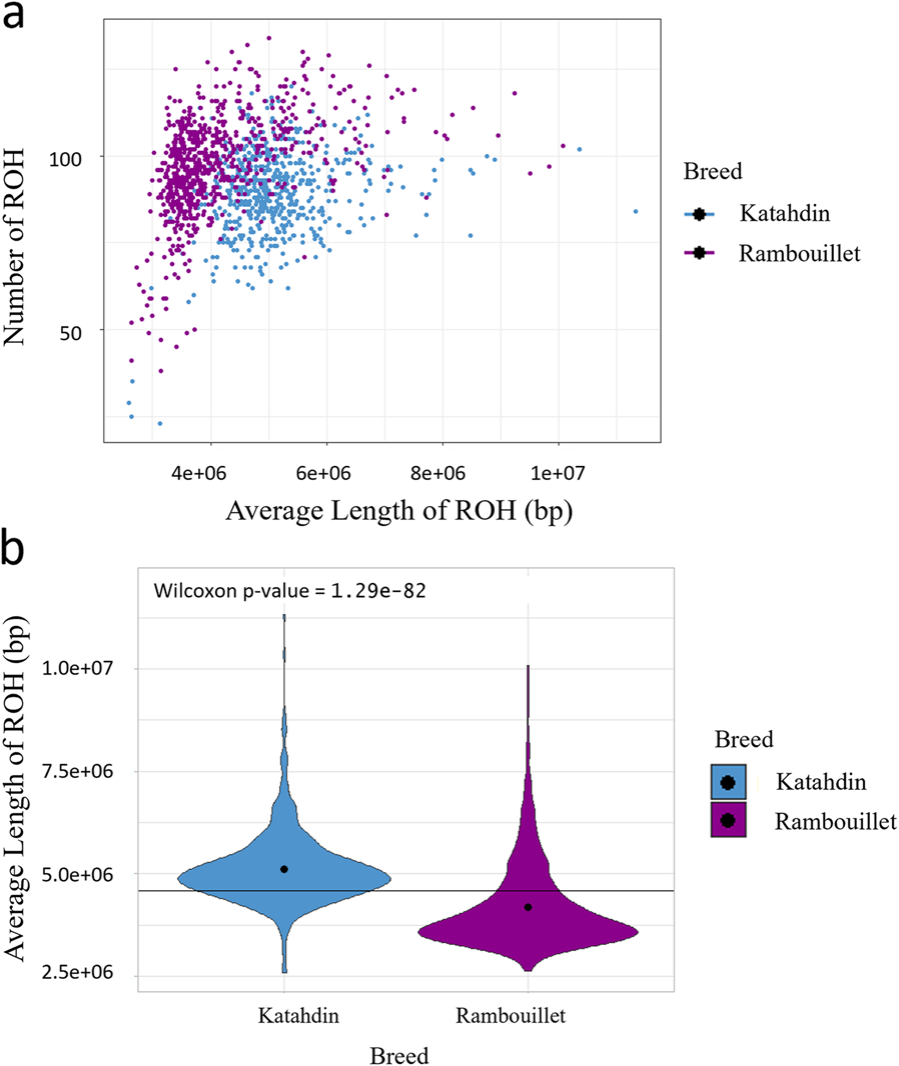
Length of ROH called by breed. **a** Plot of the average length of ROH (x-axis) by the total number of ROH called per individual (y-axis), with the Katahdin sheep plotted in blue and the Rambouillet sheep in purple; **b** Average length of ROH by breed, with the overall mean indicated by the horizontal line and breed means indicated by black points. The Wilcoxon *P*-value of 'average length ~ breed' is 1.29e−82

There were 51,992 and 72,946 ROH called for Katahdin and Rambouillet, respectively. The ROH were categorized into five classes by length, with classes defined by 0–6 Mb, 6–12 Mb, 12–24 Mb, 24–48 Mb, and > 48 Mb. The majority of the ROH identified were shorter than 6 Mb in length; 86% of the Rambouillet total ROH and 75% of the Katahdin total ROH were within this class (see Additional file 3: Table S2). The percentages of 6–12 Mb and 12–24 Mb long ROH were greater in Katahdin than in Rambouillet (see Additional file 4: Fig. S2). The average lengths of ROH called in Katahdin and Rambouillet were significantly different (Wilcoxon *P*-value = 1.29e−82), with Rambouillet having significantly shorter mean ROH than Katahdin (Fig. 3). The differences observed in mean ROH length and in percentage of ROH by class may reflect differences in LD decay at signatures of selection between these breeds. As LD breaks down rapidly over distance, short ROH can indicate more ancient inbreeding or selection events while long ROH are indicative of more recent selection [77, 78].

**Fig. 4:**
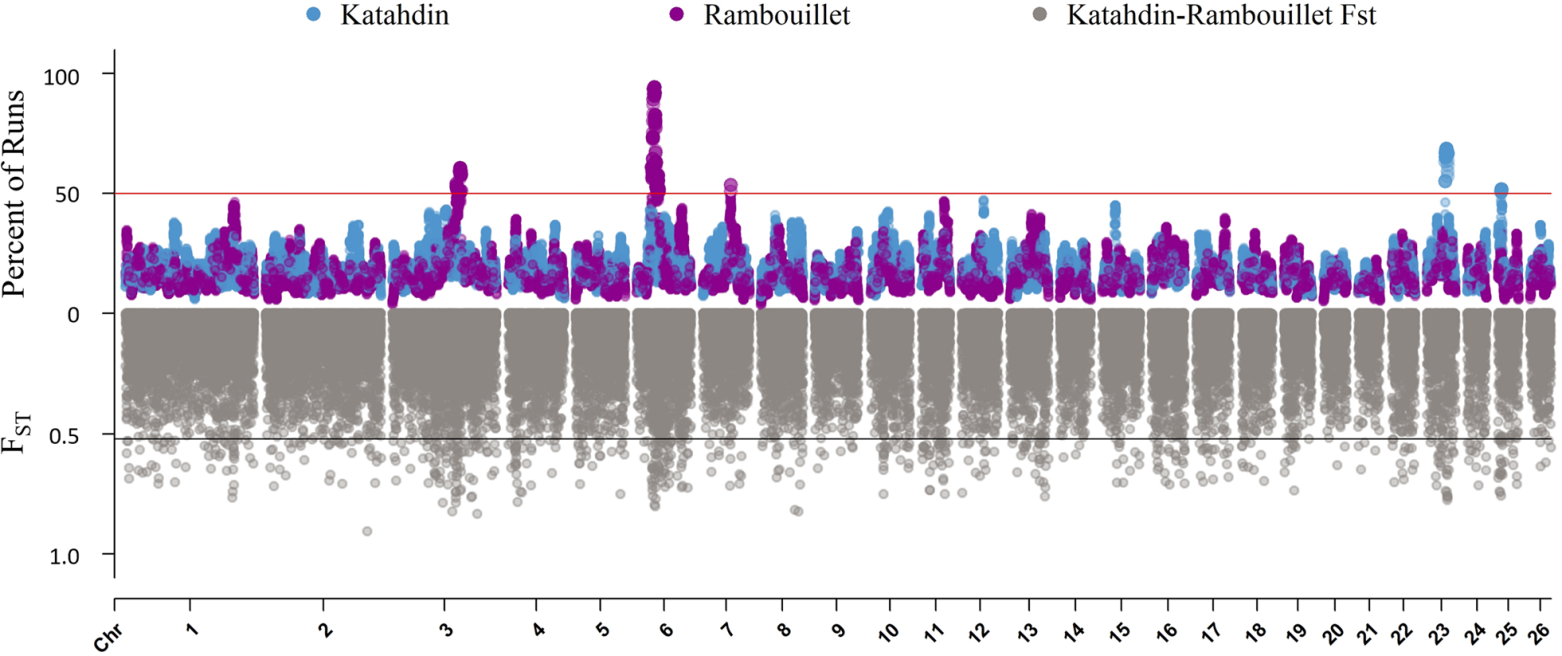
Manhattan plot of ROH and F_ST_ results for Katahdin and Rambouillet sheep. The top of the plot (scale 0 to 100) is the percentage of SNPs called in an ROH in Katahdin (blue) and Rambouillet (purple) breeds. The bottom of the plot (scale 0 to 1.0) contains the results of the Katahdin-Rambouillet pairwise F_ST_ of each SNP (gray)

**Fig. 5:**
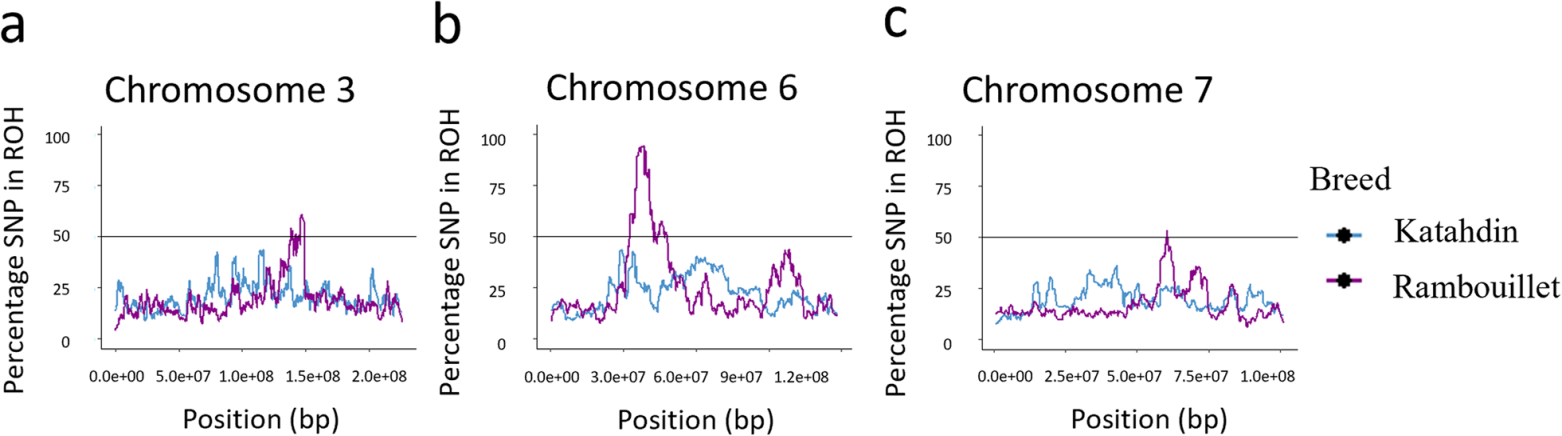
Chromosomes with ROH called in 50% or more of the Rambouillet sheep. **a** ROH called on chromosome 3; **b** ROH called on chromosome 6; **c **ROH called on chromosome 7. The ROH called in Katahdin are plotted in blue and the ROH in Rambouillet are plotted in purple

**Fig. 6:**
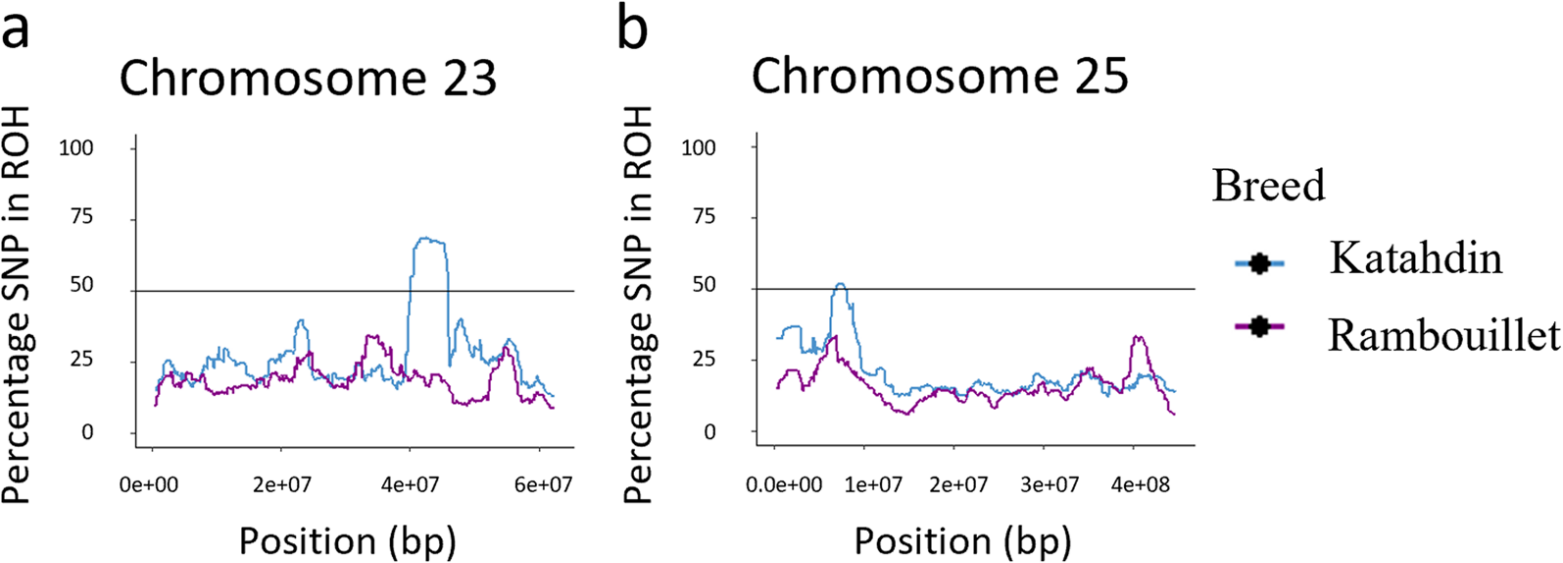
Chromosomes with ROH called in 50% or more of the Katahdin sheep. **a** ROH called on chromosome 23; **b** ROH called on chromosome 25. The ROH called in Katahdin are plotted in blue and the ROH in Rambouillet are plotted in purple

A ROH island was defined by the presence of SNPs called within a ROH in 50% or more of the animals of a breed (Fig. 4). The ROH analysis identified three ROH islands in Rambouillet and two ROH islands in Katahdin (Table 4). The ROH island identified in Rambouillet on chromosome 6 had both the largest number of SNPs called in an ROH island (202 SNPs) and the highest percentage of animals called in an ROH at the same SNP (94.23%). ROH islands in Rambouillet were identified on chromosome 3 (Fig. 5a), chromosome 6 (Fig. 5b), and chromosome 7 (Fig. 5c), and those in Katahdin on chromosome 23 (Fig. 6a) and chromosome 25 (Fig. 6b). There was some overlap between ROH islands called in Rambouillet and Dorper sheep on chromosome 6, although the ROH called in Rambouillet were both longer and more highly conserved within the breed (see Additional file 5: Table S3).

**Table 4:** Regions comprised of SNPs called within a ROH in 50% or more of the animals of a breed

Breed	Chr	Number of SNPs	AVG % SNPs in run	Max % SNPs in run	Range (bp)
Katahdin	23	50	65.82	68.67	40,163,730–45,746,963
	25	18	51.54	51.81	6,929,232–7,836,577
Rambouillet	3	71	55.70	60.67	138,563,593–148,939,496
	6	202	68.38	94.23	32,795,860–47,985,710
	7	3	52.62	53.56	60,566,239–60,647,707

Chr: chromosome number; AVG: average; Max: maximum

In Katahdin sheep, 50 SNPs were called within an ROH in 50% or more of Katahdin sheep in a region on chromosome 23 and 18 SNPs were called in a region on chromosome 25. In Rambouillet, a region on chromosome 3 contained 71 SNPs, chromosome 6 contained 202 SNPs, and chromosome 7 contained three SNPs

The genomic regions of ROH islands were evaluated for the presence of known and predicted genes. Seventy-six genes were identified in the Katahdin ROH islands, including 20 uncharacterized LOC genes without predicted *Homo sapiens* orthologs, and two copy number variants (CNV) of the *TRNAC-GCA* gene. Fifty-five unique genes were used for pathway enrichment queries (see Additional file 6: Table S4). For Rambouillet, 117 unique genes, multiple copies of tRNA genes *TRNAS-GGA*, *TRNAH-AUG*, *TRNAW-CCA*, and *TRNAC-GCA,* and 37 uncharacterized LOC genes were identified between the ROH islands on chromosomes 3 and 6 (see Additional file 7: Table S5). The ROH island identified on chromosome 7 was intergenic. Known or predicted protein–protein interactions of genes located within ROH islands were identified through query of the STRING database. For genes within the Rambouillet ROH island on chromosome 6, the largest number of interactions was found for FAM184B (9 proteins), FAM13A (7 proteins), and for CCSER1, LAP3, LCORL, MED28, and NCAPG, all of which had interactions with six proteins. Within the ROH island on chromosome 3, the largest number of interactions was found for TMEM117 (7 proteins), ZCRB1 (6 proteins), and HNRNPA1 (5 proteins). Analysis of protein–protein interactions for the Katahdin ROH island on chromosome 23 identified four interactions with NDUFV2, and analysis of the ROH island on chromosome 25 contained only interactions of ARID4B with TOMM20 and RBM34 (see Additional file 8: Table S6).

Pairwise F_ST_ of 0.140, 0.156, and 0.161 were estimated between Katahdin and Rambouillet, between Rambouillet and Dorper, and between Katahdin and Dorper, respectively. Thresholds of + 3 standard deviations above the average were calculated for each breed comparison to identify outlier SNPs that showed the greatest amount of differentiation. As expected from the pairwise estimates, the Katahdin-Rambouillet SNP comparisons showed the lowest average F_ST_ and smallest standard deviation. The F_ST_ threshold for Katahdin-Rambouillet outlier SNPs was 0.52, and for both Rambouillet-Dorper and Katahdin-Dorper comparisons, the threshold was 0.58 (Table 5). The pairwise F_ST_ statistics for each SNP in the Katahdin-Rambouillet analysis were compared against ROH island results (Fig. 4).

**Table 5:** Average F_ST_ estimates between Katahdin, Dorper, and Rambouillet breeds

	Katahdin-Rambouillet	Katahdin- Dorper	Rambouillet-Dorper
AVG	0.1149	0.1319	0.1294
SD	0.1345	0.1493	0.1506
Threshold	0.5184	0.5799	0.5813

The threshold for calling outlier values was the average (AVG) + 3 standard deviation (SD)

**Fig. 7:**
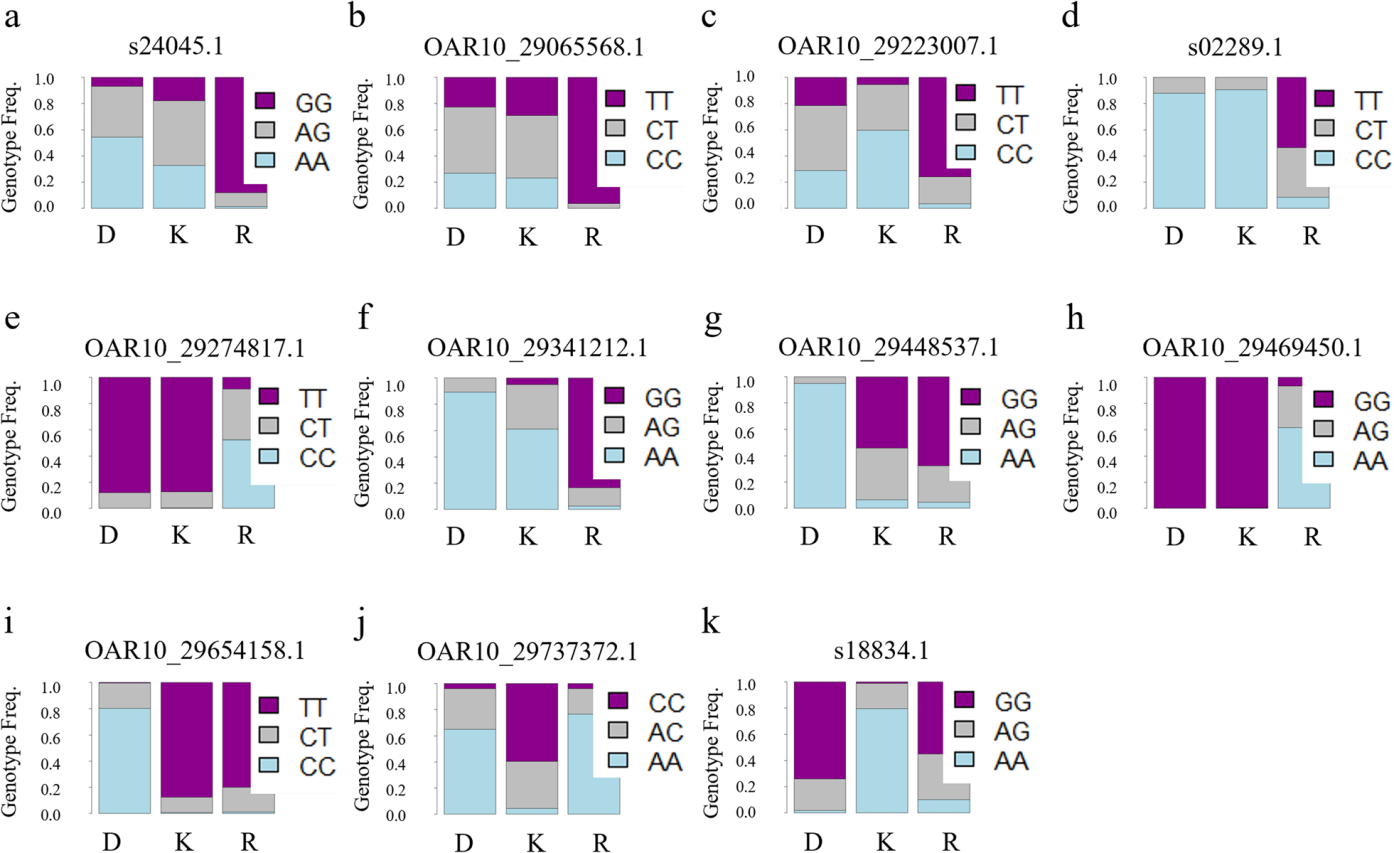
SNPs with high F_ST_ in the region of the *FRY* and *RXFP2* genes*.* **a** Stacked bar chart representing genotype frequencies at SNP s24045.1; **b** Stacked bar chart representing genotype frequencies at SNP OAR10_29065568.1; **c** Stacked bar chart representing genotype frequencies at SNP OAR10_29223007.1; **d** Stacked bar chart representing genotype frequencies at SNP s02289.1; **e** Stacked bar chart representing genotype frequencies at SNP OAR10_29274817.1; **f** Stacked bar chart representing genotype frequencies at SNP OAR10_29341212.1; **g** Stacked bar chart representing genotype frequencies at SNP OAR10_29448537.1; **h** Stacked bar chart representing genotype frequencies at SNP OAR10_29469450.1; **i** Stacked bar chart representing genotype frequencies at SNP OAR10_29654158.1; **j** Stacked bar chart representing genotype frequencies at SNP OAR10_29737372.1; **k** Stacked bar chart representing genotype frequencies at SNP s18834.1. D: Dorper; K: Katahdin; R: Rambouillet

The nearest gene of the ten SNPs with the highest F_ST_ estimates from each pairwise F_ST_ analysis was determined (Table 6). The SNP OAR2_231739122.1 had the highest F_ST_ (0.9069) in the Katahdin-Rambouillet analysis and was positioned 935 bp downstream of the *CXCR2* gene. For the Katahdin-Dorper analysis, the SNP OAR2_88734520.1 had an F_ST_ value of 0.9735 and was within the *CCDC171* gene, and for Rambouillet-Dorper, the SNP OAR1_292866363.1 had an F_ST_ value of 0.8829 and was positioned within the *METTL6* gene. Among the top ten SNPs identified in the Rambouillet-Dorper analysis, four were located within or near the *LCORL* gene. In addition, all pairwise F_ST_ comparisons identified outlier SNPs within the region of the *FRY* and *RXFP2* genes (Table 7 and Fig. 7).

**Table 6:** Genomic information for 10 SNPs with the highest F_ST_ in each pairwise breed comparison

Pairwise breed comparison	Chr:bp	F_ST_	Gene
Katahdin-Rambouillet			
OAR2_231739122.1	2:220,204,274	0.9069	935 bp downstream of *C-X-C motif chemokine receptor 2* (*CXCR2*)
OAR3_138331159.1	3:129,837,912	0.8236	24,522 bp from *mitochondrial ribosomal protein L42* (*MRPL42*)
OAR3_146778162.1	3:137,510,701	0.7882	*KAT8 regulatory NSL complex subunit 2* (*KANSL2*)
s39351.1	3:183,889,776	0.8339	8508 bp from *DENN domain containing 5B* (*DENND5B*)
OAR4_19418235.1	4:19,933,519	0.7842	1464 bp upstream of *PHD finger protein 14* (*PHF14*)
OAR6_42557643.1	6:39,000,766	0.796	207,018 bp from *protein SET-like* (LOC10110458)
OAR6_44450940.1	6:40,475,050	0.8022	*Slit guidance ligand 2* (*SLIT2*)
OAR8_78922714.1	8:73,722,766	0.8178	*TGF-beta activated kinase 1 (MAP3K7) binding protein 2 (TAB2)*
s01826.1	8:81,097,505	0.8236	65,227 bp from *AT-rich interaction domain 1B* (*ARID1B*)
OAR23_46900744.1	23:44,502,153	0.7756	3397 bp from heterogeneous nuclear ribonucleoproteins A2/B1-like (LOC101108009)
Katahdin-Dorper			
OAR2_88734520.1	2:84,038,487	0.9735	*Coiled-coil domain containing 171* (*CCDC171*)
OAR3_92705824.1	3:87,616,185	0.8486	*Cysteine rich transmembrane BMP regulator 1* (*CRIM1*)
s70203.1	4:35,066,909	0.9462	*Glutamate metabotropic receptor 3* (*GRM3*)
OAR6_64114132.1	6:58,970,273	0.8836	*KELCH like family member 5* (*KLHL5*)
OAR8_29480020.1	8:27,186,942	0.852	*methyltransferase like 24* (*METTL24*)
OAR14_25081675.1	14:24,402,748	0.8856	*Nucleoporin 93* (*NUP93*)
OAR14_53703182.1	14:51,456,105	0.9254	742 bp upstream of *zinc finger protein 575* (*ZNF575*)
s30024.1	25:6,684,555	0.8544	31,987 bp from *TAR (HIV-1) RNA binding protein 1* (*TARBP1*)
s44881.1	25:6,938,538	0.8551	27,307 bp from *small nucleolar RNA SNORA40* (LOC114110918)
OAR25_17689768.1	25:16,484,790	0.8508	49,538 bp from *transmembrane protein 26* (*TMEM26*)
Rambouillet-Dorper			
s41709.1	2:16,050,607	0.8904	48,461 bp from *zinc finger protein 462* (*ZNF462*)
OAR6_41003295.1	6:37,533,595	0.8818	1873 bp upstream of *matrix extracellular phosphoglycoprotein* (*MEPE*)
OAR6_41709987.1	6:38,197,739	0.896	*Ligand dependent nuclear receptor corepressor like* (*LCORL*)
OAR6_41768532.1	6:38,248,979	0.9007	26,028 bp from *LCORL*
OAR6_41850329.1	6:38,330,994	0.9009	108,043 bp from *LCORL*
OAR6_41925630.1	6:38,397,839	0.8971	174,888 bp from *LCORL*
s01370.1	9:27,800,948	0.8827	9194 bp from *tribbles pseudokinase 1* (*TRIB1*)
OAR14_25081675.1	14:24,402,748	0.8733	*Nucleoporin 93* (*NUP93*)
OAR16_42184944.1	16:39,117,011	0.9006	131,081 bp from *prolactin receptor* (*PR*)
OAR24_27348134_X.1	24:25,380,060	0.8806	12,165 bp from *interleukin 4 receptor* (*IL4R*)

**Table 7:** Fixation index (F_ST_) scores for SNPs in the region harboring the *FRY* and *RXFP2* genes

SNP	Gene	Chr:bp	F_ST_		
			Katahdin-Dorper	Rambouillet-Dorper	Katahdin-Rambouillet
s24045.1	*FRY, ZAR1L*	10:29,041,335	0.0531	0.6995*	0.4769
OAR10_29065568.1	*FRY*	10:29,071,493	0.0034	0.6139*	0.4614
OAR10_29223007.1	*FRY*	10:29,230,973	0.1191	0.3418	0.5809*
s02289.1	*FRY*	10:29,244,858	0.0007	0.5767	0.6366*
OAR10_29274817.1	*FRY*	10:29,281,423	− 0.0012	0.5676	0.6031*
OAR10_29341212.1	*FRY*	10:29,347,138	0.0921	0.8234*	0.6517*
OAR10_29448537.1	Downstream of *RXFP2*	10:29,458,417	0.6443*	0.7264*	0.0164
OAR10_29469450.1	Downstream of *RXFP2*	10:29,479,765	− 0.0001	0.6992*	0.7510*
OAR10_29654158.1	Upstream of *RXFP2*	10:29,654,912	0.8321*	0.7709	0.0094
OAR10_29737372.1	Upstream of *RXFP2*	10:29,753,249	0.5008	0.0115	0.5895*
s18834.1	Upstream of *RXFP2*	10:29,847,303	0.7320	0.0479	0.5521*

*Denotes that the F_ST_ score is above the breed comparison threshold of either 0.58 (Katahdin-Dorper and Rambouillet-Dorper) or 0.52 (Katahdin-Rambouillet) and is significant by Fisher's exact test

In total, 554 outlier SNPs were identified in the Katahdin-Rambouillet F_ST_ analysis. Of these 554 SNPs, 157 were positioned within 200,000 bp of at least one other outlier SNP, which together defined 63 F_ST_ regions containing 233 unique and characterized genes (see Additional file 9: Table S7). These genes included the tRNA genes *TRNAC-GCA* (four CNV), *TRNAW-CCA* (three CNV), *TRNAE-CUC* (two CNV), and *TRNAS-GGA* (one CNV). The F_ST_ regions were in part consistent with the ROH results, with seven SNPs being identified through both the Katahdin ROH and Katahdin-Rambouillet outlier F_ST_ analyses, and 18 SNPs being identified through both the Rambouillet ROH and Katahdin-Rambouillet outlier F_ST_ analyses (see Additional file 10: Table S8). The GO pathway enrichment analysis of these genes identified significant enrichment for biological processes including regulation of cellular glucuronidation and glucuronosyltransferase activity, and significant enrichment for the molecular function pathway UDP-glycosyltransferase activity (see Additional file 11: Table S9). KEGG Mapper pathway analysis revealed genes that are involved in many pathways, including, among others, B cell receptor signaling, taste transduction, circadian rhythm, cytokine-cytokine receptor interaction, endocytosis, growth hormone synthesis/secretion/action, hematopoietic cell lineage, HIF-1 signaling, IL-17 signaling, and longevity regulation pathways. In addition, there were a number of pathways related to viral infection, such as herpes simplex virus 1, human immunodeficiency virus 1, and human cytomegalovirus (see Additional file 12: Table S10).

Analyses with the Dorper breed were used to better understand allelic differentiation in the Katahdin and Rambouillet breeds. All F_ST_ regions were analysed to identify the genes that were present in both pairwise comparisons of a breed: for example, genes identified in both the Rambouillet-Dorper and Katahdin-Rambouillet F_ST_ analyses were further explored to give context to the F_ST_ signals associated with the Rambouillet. Fifty-one genes were in common for the Rambouillet, 31 for the Katahdin, and 21 for the Dorper comparisons (Table 8). Many of these genes were previously identified in studies on signatures of selection or are candidate genes for production and/or health traits in sheep. The genes reported in Table 8 for each breed were queried through KEGG. These analyses identified pathways for metabolic pathways, olfactory transduction, and cytokine-cytokine receptor interaction (Table 9). In addition, genes in common between ROH islands and F_ST_ regions were evaluated for Katahdin and Rambouillet breeds (Table 10).

**Table 8:** Genes that were in common between F_ST_ comparisons

Breed (number of genes)	Genes within all F_ST_ analyses of a breed [reference]
Rambouillet (51)	*ADIRF* [79], *BMPR1A* [80], *CCSER1* [81], *CSF3* [82], EEF1A1* [83], EIF2S2* [84], *EVI2A* [85], *EVI2B* [85], *FAM25A*, FRY* [86], *GSDMA* [87], *GSDMB*, *LDB3* [88], *LRRC3C*, *MED24*, *MMRN2*, *MRPS18C*, *MSL1*, *MTIF2*, *NF1* [89], *NR1D1* [90], *OMG* [91]*, OPN4*, *OR10A2*, *OR10A5*, *OR2AG1* [92], *OR2D3*, *OR8S1*, *ORMDL3*, *PCED1B*, *PSMD3* [93], *RAB11FIP4* [94], *RASGEF1B*, *RPL10A* [95], *RPL6* [96], RXFP2* [83], *SCAMP2*, *SLC38A1*, *SLC38A2*, *SNCG* [97], *SNORD124*, *THRA* [93], *TMA7*, *TNFSF18* [98], *TRNAC-ACA*, TRNAC-GCA* [99], TRNAG-UCC*, TRNAS-GGA* [100], TRNAW-CCA* [101], *UAP1*, *ZPBP2*
Katahdin (31)	*ADCY6* [102], *B3GALNT2*, *B3GLCT*, *CACNB3* [103], *CCNT1* [91], *DDX23*, EEF1A1* [83], EIF2S2* [84], *FBXL14*, FRY* [86], *INPP4A*, *IRF2BP2*, *KANSL2* [104], *LALBA* [97], *MGAT4A, MRPL42* [105], *NUDT4* [105], *RND1*, RXFP2* [83], *SNORA40, SOCS2* [106], *ST13*, *TARBP1*, *TEX49*, TRNAC-GCA* [99], *TRNAE-CUC*, TRNAG-UCC*, TRNAS-GGA* [100], TRNAW-CCA* [101], *UBE2N* [107], *UNC50*
Dorper (21)	*ANO6* [108], *ATXN7L3B*, *DIP2C* [81], EEF1A1* [83], EIF2S2* [84], *EPB41L4B* [109], FRY* [86], *GRIN2B* [110], *MFF* [111], *PTPN3* [112], RXFP2* [83], *SLIT2* [113], *SNORD31*, TRNAC-GCA* [99], *TRNAG-CCC*, TRNAG-UCC*, *TRNAH-AUG*, *TRNAS-GCU*, TRNAS-GGA* [100], TRNAW-CCA* [101], *ZMYND11* [114]

Genes that were present in all F_ST_ comparisons of a breed (e.g., for Rambouillet, genes that were present in both the Katahdin-Rambouillet and Rambouillet-Dorper comparisons). The list numbers of the relevant literature references for genes identified as candidate genes or within signatures of selection for production and/or health traits in sheep are in brackets. *indicates that the gene is present in more than one breed list. tRNA genes may be members of the same family located at multiple loci between breeds or analyses

**Table 9:** KEGG results for genes which were in common between both comparisons of Rambouillet or Katahdin

Breed	KEGG Mapper pathway (number of genes)	Enriched genes
Rambouillet	Olfactory transduction (5)	*OR2D3*, *OR10A5*, *OR2AG1*, *OR10A2*, *OR8S1*
	Ribosome (3)	*RPL10A*, *MRPS18C*, *RPL6*
	Cytokine-cytokine receptor interaction (3)	*CSF3*, *BMPR1A*, *TNFSF18*
	Coronavirus disease—COVID-19 (3)	*CSF3*, *RPL10A*, *RPL6*
Katahdin	Metabolic pathways (3)	*ADCY6*, *B3GALNT2*, *LALBA*

Genes present within the Rambouillet F_ST_ analyses (i.e. the Katahdin-Rambouillet and Rambouillet-Dorper F_ST_ comparisons) or the Katahdin F_ST_ analyses (i.e. the Katahdin-Rambouillet and Katahdin-Dorper F_ST_ comparisons) were queried through KEGG Mapper to identify pathways of interest. The Dorper sheep did not have KEGG mapper pathways enriched with three or more gene search terms

**Table 10:** Genes that were called within the ROH islands and pairwise F_ST_ comparison regions

Breed	Genes in common between ROH and F_ST_ regions
Katahdin	*ARID4B*, *ATMIN*, *B3GALNT2*, *GGPS1*, *GNG4*, *LYST*, *RBM34*, *TBCE*, *TOMM20*, *TRNAC-ACA*, *TRNAC-GCA*, *TRNAR-CCU*, *TRNAW-CCA*
Rambouillet	*ADGRA3*, *AMIGO2*, *ANO6*, *CCSER1*, *COL2A1*, *DCAF16*, *EIF2S3*, *FAM184B*, *GBA3*, *GRID2*, *HDAC7*, *KCNIP4*, *LAP3*, *LCORL*, *LRRK2*, *MED28*, *MFF*, *NCAPG*, *PCED1B, RAN*, *RPL10A*, *RPL37*, *SLC2A13*, *SLC38A1*, *SLC38A2*, *SLC38A4*, *SLIT2*, *SNCA*, *STIM2*, *TBC1D19*, *TMA7*, *TMEM106C*, *TRNAA-CGC*, *TRNAC-GCA*, *TRNAG-CCC*, *TRNAH-AUG*, *TRNAS-GGA*, *TRNAW-CCA*, *VDR*

The Katahdin genes are present in the Katahdin ROH islands as well as the F_ST_ region(s) between Katahdin-Rambouillet and/or Katahdin-Dorper, the Rambouillet genes are present in Rambouillet ROH islands and the F_ST_ region(s) between Katahdin-Rambouillet and/or Rambouillet-Dorper

The Katahdin-Dorper analysis identified 513 outlier SNPs, including 109 SNPs located near to each other, which defined 49 regions. The 144 genes contained within these regions were used for pathway analysis. Genes in these regions were part of KEGG pathway terms, including chemokine signaling and cytokine-cytokine receptor interaction (see Additional file 13: Table S11). In the Rambouillet-Dorper analysis, 569 outlier SNPs were identified, with 167 SNPs defining 73 F_ST_ regions. In total, 264 genes were located within these regions. Some of the pathways identified by KEGG Mapper analysis included biosynthesis of unsaturated fatty acids, neutrophil extracellular trap formation, olfactory transduction (with nine related genes), and prion disease (see Additional file 14: Table S12). The pathway analyses of the Dorper ROH results are provided as supplementary material (see Additional file 15: Table S13; Additional file 16: Table S14; Additional file 17: Table S15; Additional file 18: Table S16). In addition, the outlier F_ST_ results described here from the Weir and Cockerham model implemented in Plink were compared against the results of a BayeScan F_ST_ analysis (see Additional file 19: Table S17).

Protein–protein interactions were identified for genes implicated by pairwise F_ST_ analyses with STRING. Query with the genes from the Katahdin-Rambouillet F_ST_ outlier regions revealed the largest number of interactions with ribosomal proteins RPL23A (19 proteins), RPL5 (18 proteins), and RPL10A, RPL6, and RPL7, each with 15 protein–protein interactions. Query with the genes from the Rambouillet-Dorper F_ST_ outlier regions identified 19 protein–protein interactions with RPL8, 18 interactions with RPL6, and 17 interactions each with RECQL4 and RPL10A. The largest number of interactions in the Katahdin-Dorper F_ST_ outlier regions were with EIF4A3, KCNQ1, NPM1, and TSSC4, all of which had seven protein–protein interactions (see Additional file 8: Table S6).

## Discussion

In this study, we analyzed the genetic diversity and signatures of selection of Rambouillet, Katahdin, and Dorper sheep through within-breed (ROH approach) and pairwise breed (F_ST_ outlier approach) comparisons. The results reported here concern genomic regions that are under selection in the considered U.S. sheep. In addition, this study identified commonalities with previously identified signatures of selection from a diverse range of breeds, which allows our findings to contribute to a broad conversation about the signatures of selection and breed diversity of worldwide sheep.

A landmark paper in sheep genetic diversity research was published in 2012 by Kijas and colleagues [115]. Their study analyzed population structure and signatures of selection of 2819 sheep sampled from 74 breeds, including 102 Rambouillet sheep. The inbreeding coefficient (F) and *N*_*e*_ reported for these Rambouillet sheep were 0.14 and 709, respectively. These statistics are quite different from those of the Rambouillet analyzed in the current study. While a larger number of Rambouillet (N = 745) were sampled in this paper, the average inbreeding coefficient (F_ROH_) was found to be higher (0.169) and the current *N*_*e*_ was smaller (NeEstimator: 56.9, GONe: 111.8) than those reported previously [115]. It is possible that these differences result from differences between the F statistic and *N*_*e*_ calculation methods. A previous study found that two genotype-based inbreeding coefficients (F_SNP_ and F_ROH_) had correlation coefficients ranging from 0.78 to 0.88, indicating that while related, the degree of inbreeding is not entirely comparable between subcategories of F statistics [116]. The results of the current study found that F and F_ROH_ showed different levels of inbreeding, with pairwise breed differences being most significant in F_ROH_ estimates. The *N*_*e*_ reported in our study are estimated for current and historic generations while the *N*_*e*_ estimates reported previously by Kijas and colleagues [115] are not described in terms of generational distance and are therefore difficult to compare directly.

A previous study was conducted with genotype data from 4884 Katahdin sheep [72]. The 581 Katahdin sheep used in our study were also analyzed as part of this larger population, although the genotype data used between these publications were collected from separate platforms. In spite of the difference in sample sizes between these studies, the SNeP *N*_*e*_ estimations 13 generations ago were very similar (*N*_*e*_ of 172 or 178 estimated from 4884 or 581 sheep, respectively). This agreement between *N*_*e*_ estimations supports the validity of these estimates.

The *N*_*e*_ estimates reported between the SNeP, GONe, and NeEstimator approaches varied. Based on SNeP, Rambouillet had consistently larger *N*_*e*_ than Katahdin, and both breeds showed a decline in *N*_*e*_ between each subsequent generation. This ranking of the Rambouillet and Katahdin breeds was also observed in the historic *N*_*e*_ reported through GONe, although in more recent generations there were multiple reranking events in which the *N*_*e*_ of Katahdin exceeded that of Rambouillet, and in the current generation, both NeEstimator and GONe calculated a larger *N*_*e*_ for Katahdin than for Rambouillet. However, Katahdin had significantly higher average F and F_ROH_ compared to Rambouillet, which suggests comparatively less current breed diversity. While the largest and most conserved ROH was identified in Rambouillet, Katahdin had a greater proportion of ROH within the larger 6–12 Mb class. The presence of more ROH of greater length suggests recent inbreeding events in the history of the Katahdin breed. Taken together, these results support the importance of evaluating evidence from multiple inbreeding and *N*_*e*_ models, since relying on a single estimate might bias interpretation of genetic diversity.

The largest ROH Island was positioned between 32.8 and 48.0 Mb on chromosome 6 and encompassed 64 unique genes. This region was previously reported to be under selection in sheep and cattle [23, 117–120] and many of the associated genes were suggested as candidates for production traits. In cattle, the *ABCG2, IBSP*, *PIGY*, *PKD2,* and *MEPE* genes have been associated with yearling weight [121]; *NCAPG*, *LCORL,* and *LAP3* with body weights and calving ease [122]; and *ABCG2*, *LAP3*, *NCAPG*, *DCAF16,* and *LCORL* with milk total solid percentage [123]. Similarly in sheep, quantitative trait loci (QTL) associated with the *DCAF16*, *LAP3*, *LCORL*, *NCAPG, NAP1L5,* and *PPARGC1A* genes have been reported for somatic cell score or milk yield in dairy sheep [124]; *DCAF16, LCORL,* and *NCAPG* genes associated with body weight [125]; *SPP1* and *LAP3* genes associated with weight [126]; *LCORL* associated with meat productivity [127]; *SLIT2* and *ABCG2* genes associated with fat deposition and milk production, respectively [115, 128]; and genes *HERC3, HERC5, HERC6, IBSP,* and *SPP1* associated with parasite resistance [129]. In the current study, the ROH island identified in this region was highly conserved, with more than 94% of the Rambouillet animals having a ROH called. Rambouillet sheep are raised for their carcass and wool characteristics and, as a breed, are generally considered to be susceptible to parasite infection [130]. The ROH island in this region may be the result of past selection for weight or carcass traits and the homozygosity at genes relating to milk yield or parasite resistance may have occurred through hitchhiking [131].

Similarly, the ROH island identified on chromosome 3 contains genes that were previously described within signatures of selection in goats and cattle, including *ADAMTS20*, *GXYLT1*, *IRAK4*, *PRICKLE1*, *PUS7L*, *TWF1*, *YAF2*, and *ZCRB1* [132–134]. Expression of *ADAMTS20* has been reported to be upregulated in ewes with endometritis [135], and members of the *ADAMTS* family have been associated with divergent prolificacy of sheep and goats [136]. In goats, the *ADAMTS20* gene has also been associated with coat color [137]. The *IRAK4* gene encodes interleukin-1 receptor-associated kinase 4, a key regulator of innate immune signaling responses [138]. In addition, the *GXYLT1*, *PRICKLE1*, *YAF2*, and *ZCRB1* genes have been associated with resistance/susceptibility to *Mycobacterium avium* spp. *paratuberculosis* infection (responsible for Johne’s disease) in Holstein cattle [139]. An association study in dogs identified *TWF1* as a candidate gene for deafness, and this gene has potential roles in hair-bundle development and melanocyte dendricity [140]. Both *IRAK4* and *TWF1* were implicated as genes of interest by Tajima’s D analysis with two strains of Qinchuan cattle [133]. The occurrence of these genes within signatures of selection in other species and the associations with reproduction, coat color, and animal health suggest potential traits which may be under selection in the Rambouillet breed.

Genes that were previously reported in signatures of selection in worldwide sheep breeds were identified through F_ST_ analysis of the U.S. Rambouillet, Katahdin, and Dorper sheep in the current study. These genes include *NF1*, a negative regulator of the ras pathway (identified in the Katahdin-Rambouillet and Rambouillet-Dorper F_ST_ comparison), *OR2AG1* (identified in the Katahdin-Rambouillet and Rambouillet-Dorper F_ST_ comparison, and other olfactory receptor genes identified in all F_ST_ comparisons); and *RXFP2*, associated with horn phenotypes in sheep (present in all F_ST_ comparisons), which were reported by Kijas et al. [115]. A number of the genes identified in both F_ST_ comparisons with Katahdin (Katahdin-Rambouillet and Katahdin-Dorper) have been associated with milk production or resistance and susceptibility for mastitis. These genes included *CCNT1*, previously associated with milk and cheese-making traits [91]; *LALBA*, associated with milk production traits [141]; *MRPL42*, a candidate gene for mastitis resistance [105]; and the gene suppressor of cytokine signaling 2 (*SOCS2*) associated with mastitis susceptibility [106, 142]. Genes present in both Rambouillet F_ST_ comparisons (Katahdin-Rambouillet and Rambouillet-Dorper) have been shown to be connected with wool growth, including: *FRY*, involved in wool development through a previous F_ST_ analysis [78]; *NR1D1*, found to be differentially expressed and differentially methylated at different stages of hair follicle development [90]; *TNFSF18*, a candidate gene for staple length and fiber diameter traits [98]; and *RXFP2*, which may be linked to hair follicle growth through the cAMP synthesis pathway [143]. The *NF1*, *EVI2A*, *EVI2B,* and *OMG* genes were also identified in both of the Rambouillet F_ST_ analyses and were previously associated with adaptive response to physical exhaustion and fat deposition [85].

Copies of the *EIF2S2* gene were identified in F_ST_ regions on chromosomes 13 and 25 in the Rambouillet-Dorper and Katahdin-Rambouillet F_ST_ analyses, respectively. The copy-number variant of *EIF2S2* on chromosome 25 is caused by retrotransposition (retroCNV), which results in an insertion of the *EIF2S2* retrogene into the 3′ UTR of *IRF2BP2*. This insertion has been found to be responsible for the “wooly” fleece phenotype of modern sheep [84, 144]. As a hair breed, the coats of Katahdin sheep are more similar to those of ancestral sheep breeds, in which an inner coat of fine wooly fibers lays below an outer coat comprised of hair fibers. Modern wooly sheep, such as the Rambouillet, lack the double coat of hair and ancestral sheep breeds, and instead possess a single coat comprised of wooly fibers of mostly uniform dimension [84]. It is unclear what is the biological mechanism that is responsible for Rambouillet having differentiation in the regions of both the retroCNV and the *EIF2S2* gene, but the high F_ST_ in the region on chromosome 25 is likely related to differences between hair and wool coat types.

The ROH island identified on chromosome 25 in the Katahdin sheep overlapped partly with the Katahdin-Rambouillet F_ST_ region containing the *IRF2BP2* gene. The F_ST_ region ranged from 6.6 to 7.0 Mb while the ROH island ranged from 6.9 to 7.8 Mb, creating an overall signature of selection encompassing a region from 6.6 to 7.8 Mb on chromosome 25. Three SNPs positioned at, respectively, 6.94 Mbp, 7.67 Mbp, and 7.73 Mbp, were identified by both the ROH and F_ST_ outlier analyses. This region contains the *TOMM20*, *RBM34*, *ARID4B*, *GGPS1*, *TBCE*, *B3GALNT2*, *ATMIN*, *GNG4*, and *LYST* genes, which were previously implicated in tail fat deposition, the ancestral-like coarse wool phenotype, coat color regulation, and response to *Brucella ovis* infection [145–149]. The identification of this signature of selection through both F_ST_ and ROH analyses, which encompasses genes that are important for both immune pathways and hair type, suggests a potential relationship between the genetic control of these traits in Katahdin sheep.

The ROH island identified on chromosome 23 in Katahdin sheep harbors genes compiled in KEGG immune pathways, including the *LAMA1* gene in the ECM-receptor interaction, toxoplasmosis, and viral myocarditis pathways; *TUBB6* in the phagosome, *Salmonella* infection and pathogenic *E. coli* infection pathways; and *PTPRM* in the adherens junction and cell adhesion molecules pathways. In a study with Scottish Blackface lambs, the *LAMA1* gene was found to be divergently expressed between lambs with low versus high fecal egg count phenotypes [150]. The *PTPRM* gene has been described to have a role in the telogen phase of hair follicle growth in Dorper sheep [151], and in studies with humans and biomedical models, it has been shown to be expressed by T cells and to have a dysregulated expression in patients with immune-mediated skin disease [152, 153]. While there is no current evidence for a specific immune role for *TUBB6* in sheep, transcription of *TUBB6* and other phagosome-associated genes were found to be downregulated in the pituitary gland of nutrient-restricted ewes during late gestation [154], and enhanced transcription was identified during the late phase of *Eimeria bovis* infection in culture with host bovine cells [155].

The SNP with the highest F_ST_ score for the Katahdin-Rambouillet analysis was OAR2_231739122.1, which is positioned 935 bp downstream of the *CXCR2* gene. This gene encodes the principal membrane-bound chemokine receptor that is responsible for mediating neutrophil recruitment [156]. In sheep, *CXCR2* has been implicated in clinical mastitis and resistance to gastrointestinal nematode infection [157, 158]. A number of immune-related pathways were revealed through KEGG Mapper pathway analysis of the genes present within the Katahdin-Rambouillet F_ST_ regions. These pathways included leukocyte transendothelial migration (*AFDN* gene), B cell receptor signaling (*CD79B* gene), cytokine-cytokine receptor interactions (*CSF3, GH1, BMPR1A, GDF5, TNFSF18* genes), IL-17 signaling (*CSF3* gene), inflammatory mediator regulation of TRP channels (*ADCY6, NTRK1, TRPM8* genes), and NF-kappa B signaling (*BCL2L1, MAP3K14* genes), among others. The high level of F_ST_ associated with these genes and pathways may reflect differences in allele frequencies contributing to the more robust immune response attributed to Katahdin sheep [33, 157].

Through KEGG Mapper pathway analysis for Katahdin-Dorper F_ST_ regions, pathways including chemokine signaling (*ADCY6*) and cytokine-cytokine receptor interaction (*TNFRSF13B*), NOD-like receptor signaling (*NLRP3* and *PANX1*), B cell receptor signaling (*CD81*), and phagosome pathways (*STX7*) were identified. These results are of particular interest in light of the divergent immune abilities of Dorper and Katahdin sheep [159, 160]. In addition, KEGG cytokine-cytokine receptor interaction pathways were identified in all F_ST_ comparisons, although the genes involved differed between analyses (see Additional file 20: Fig. S3). In the Katahdin-Rambouillet comparison, the *CSF3, GH1, BMPR1A, GDF5,* and *TNFSF18* genes were identified; in the Rambouillet-Dorper comparison, the *IL31RA, CSF3, IL17RA, GHR, IL6ST, BMPR1A,* and *TNFSF18* were identified; and in the Katahdin-Dorper comparison, only the *TNFRSF13B* was identified, as previously stated. About half of the pathway genes overlapped between the Rambouillet comparisons (*CSF3, BMPR1A,* and *TNFSF18*), suggesting that these F_ST_ regions were most strongly associated with differentiation of allele frequencies in Rambouillet sheep. There were no overlapping genes between the two Katahdin comparisons, which suggests that the greatest amount of differentiation between Katahdin and Rambouillet and between Katahdin and Dorper occur in different regions of the cytokine-cytokine receptor interaction pathways. These F_ST_ results may have a role in the overall immune response of Katahdin sheep compared to the Rambouillet and Dorper breeds that are susceptible to parasites.

The sheep used in this study were not phenotyped for coat color or the presence or absence of horns or scurs. However, Rambouillet are well-known for their light-colored coat [161], with variation being observed in different propensities for yellowing rather than pigment [162]. Dorper sheep have a white body and a black head and neck, while White Dorper sheep have an entirely white coat [163]. The Dorper analyzed in this study were from a flock founded by both Dorper and White Dorper sheep and showed a variety of color patterns, including some animals with black heads and/or black spotting and others with solid white coats. Katahdin sheep do not have a definite coat color and may have a variety of colors and patterns [164]. These breed-specific differences in coat color may have influenced the identification of signatures of selection that contain genes associated with coat pigment in the present study. Horn phenotypes are also expected to differ between these breeds. Many Katahdin producers prefer their sheep to be polled, although scurred and horned animals are permitted by breed standards [164]. The horn/polled phenotype in Rambouillet has been described as strictly sex-linked [165]. Rams in the African Dorper breed are variable and can have horns, scurs, or be polled, while females are scurred or polled [165]. These known differences may explain why multiple F_ST_ outlier SNPs were detected near the *RXFP2* gene in all pairwise comparisons of this study.

## Conclusions

This study provides insights into the signatures of selection and genetic diversity of three popular U.S. sheep breeds. The results described here support previous reports on genes that underlie signatures of selection in sheep and provide additional insights into the biological differences between Rambouillet, Katahdin, and Dorper sheep. The results of the F_ST_ analyses indicated strong population differentiation associated with genes relevant to milk production in Katahdin sheep and wool growth in Rambouillet sheep. A large and highly conserved ROH island was identified in Rambouillet sheep that contained genes of known importance for growth and carcass traits, including *LCORL* and *NCAPG*. Signatures of selection were identified in genes relevant to ancestral versus wooly coat types (*IRF2BP2*, retroCNV *EIF2S2*) and horn/polled phenotypes (*RXFP2*) in addition to many genes involved in immune-related pathways. These findings likely have relevance to the variations in physical appearance and parasite resistance ability of these breeds. Further analysis of these F_ST_ and ROH signatures of selection may provide greater insight into the selection pressures being exerted on these important breeds in the U.S. sheep industry.

### Supplementary Information

**Additional file 1: Figure S1.** Principal component analysis with Rambouillet, Katahdin, and Dorper sheep.

**Additional file 2: Table S1.** Effective population size (*N*_*e*_) for Dorper sheep. The rate of change (m) between each pair of generations is reported, with the overall rate of change reported in italics.

**Additional file 3: Table S2.** Percentage of ROH and mean size of ROH by breed and by Mbp class.

**Additional file 4: Figure S2.** Stacked bar plot for the proportion of ROH called within each ROH size class by breed.

**Additional file 5: Table S3.** ROH islands identified in Dorper sheep. ROH islands are defined by SNPs called within a ROH for more than 50% of Dorper sheep. *indicates these regions have overlap between Dorper and Rambouillet ROH calls.

**Additional file 6: Table S4.** Results of KEGG Mapper Pathway analysis of genes located within Katahdin ROH islands.

**Additional file 7: Table S5.** Results of KEGG Mapper Pathway analysis of genes located within Rambouillet ROH islands.

**Additional file 8: Table S6.** Results of STRING protein–protein interactions for genes contained within F_ST_ outlier regions and within-breed ROH islands. This excel file Table S6 contains seven sheets i.e. Sheet 1 entitled “Katahdin-Rambouillet F_ST_ STRING interactions”, Sheet 2 entitled “Rambouillet-Dorper F_ST_ STRING interactions”, Sheet 3 entitled “Katahdin-Dorper F_ST_ STRING interactions”, Sheet 4 entitled “Katahdin ROH STRING interactions, chromosome 23”, Sheet 5 entitled “Katahdin ROH STRING interactions, chromosome 25”, Sheet 6 entitled “Rambouillet ROH STRING interactions, chromosome 3” and Sheet 7 entitled “Rambouillet ROH STRING interactions, chromosome 6”.

**Additional file 9: Table S7.** Summary of F_ST_ regions by chromosome and breed comparison.

**Additional file 10: Table S8.** List of SNPs identified from both Katahdin-Rambouillet F_ST_ analysis and Katahdin or Rambouillet ROH islands.

**Additional file 11: Table S9.** Results of GO enrichment analysis of genes located within Katahdin-Rambouillet F_ST_ regions.

**Additional file 12: Table S10.** KEGG Mapper pathway results for query of Katahdin-Rambouillet genes against the *Homo sapiens* reference database.

**Additional file 13: Table S11.** KEGG Mapper pathway results for query of Katahdin-Dorper genes against the *Homo sapiens* reference database.

**Additional file 14: Table S12.** KEGG Mapper pathway results for query of Rambouillet-Dorper genes against the *Homo sapiens* reference database.

**Additional file 15: Table S13.** Most significantly enriched GO biological process function terms from Dorper ROH islands. Gene names were searched against the *Bos taurus* reference database. Each term in italics represents the most specific subclass with related parent terms directly below.

**Additional file 16: Table S14.** Significantly enriched GO cellular component terms from Dorper ROH islands. Gene names were searched against the *Bos taurus* reference database. Each term in italics represents the most specific subclass with related parent terms directly below.

**Additional file 17: Table S15.** Significantly enriched GO cellular molecular function terms from Dorper ROH islands. Gene names were searched against the *Bos taurus* reference database. Each term in italics represents the most specific subclass with related parent terms directly below.

**Additional file 18: Table S16.** KEGG Mapper pathway enrichment for genes located within Dorper ROH islands. KEGG query was made against the *Homo sapiens* reference.

**Additional file 19: Table S17.** Comparison of BayeScan and Plink F_ST_ results for each pairwise analysis. This excel Table S17 contains three sheets i.e. Sheet 1 entitled “Comparison of BayeScan and Plink F_ST_ results for the Katahdin-Rambouillet pairwise analysis”, Sheet 2 entitled “Comparison of BayeScan and Plink F_ST_ results for the Rambouillet-Dorper pairwise analysis”, and Sheet 3 entitled “Comparison of BayeScan and Plink F_ST_ results for the Katahdin-Dorper pairwise analysis”.

**Additional file 20: Figure S3.** Genes belonging to cytokine-cytokine receptor interaction pathways identified from breed F_ST_ analyses. Genes identified in Katahdin-Dorper F_ST_ analysis are in blue, genes identified from the Katahdin-Rambouillet F_ST_ analysis are in orange, and genes identified from the Rambouillet-Dorper F_ST_ analysis are in gray. Pathway figure is modified from Kanehisa Laboratories (KEGG).

## Data Availability

All relevant data are included in the manuscript and its additional files. The datasets used and analyzed during the current study are available from the corresponding author upon reasonable request.
