# Supplementary figures and images for: Genetic diversity of United States Rambouillet, Katahdin and Dorper sheep
Source: Genet Sel Evol. 2024 Jul 30;56:56. doi: 10.1186/s12711-024-00905-7 (PMC11290166; doi:10.1186/s12711-024-00905-7)

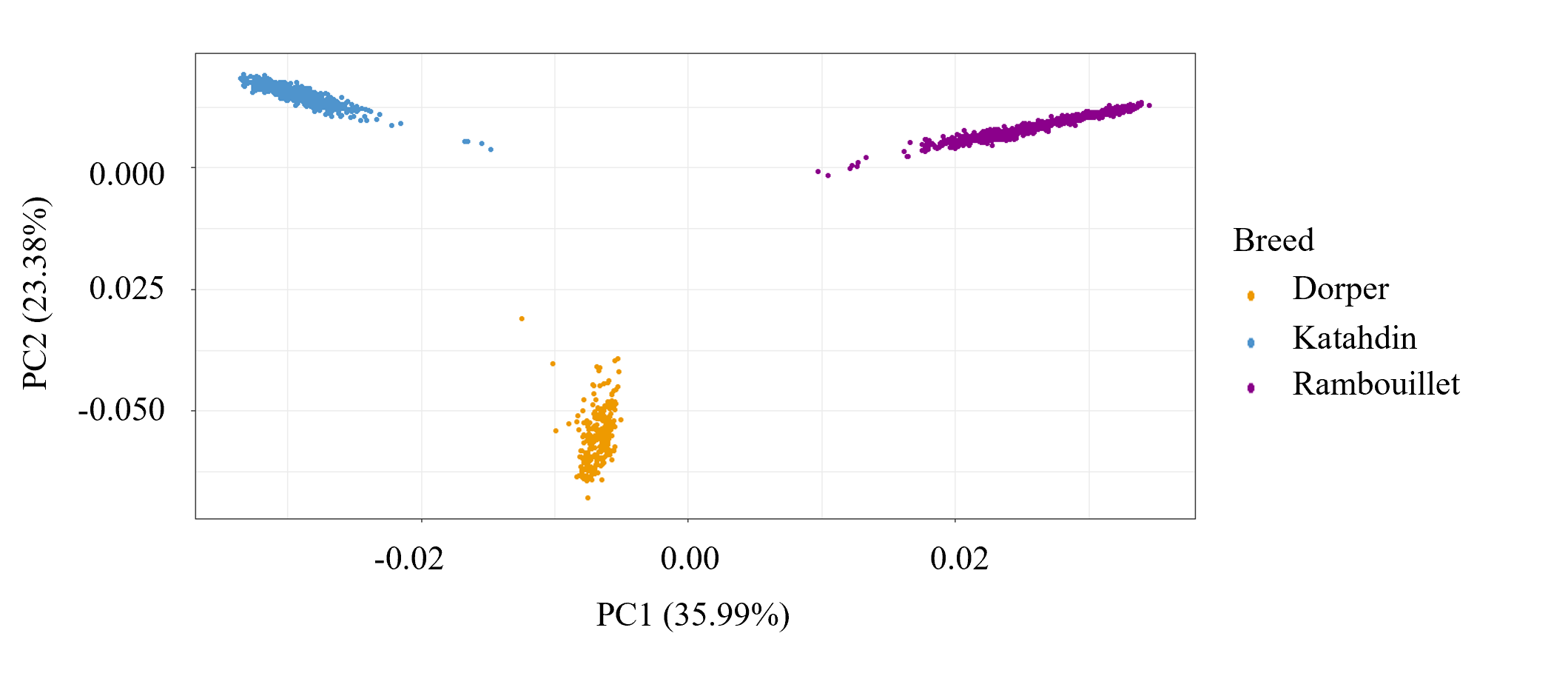

Supplement: Supplementary file 1 — Additional file 1: Figure S1. Principal component analysis with Rambouillet, Katahdin, and Dorper sheep. [file 12711_2024_905_MOESM1_ESM.png]

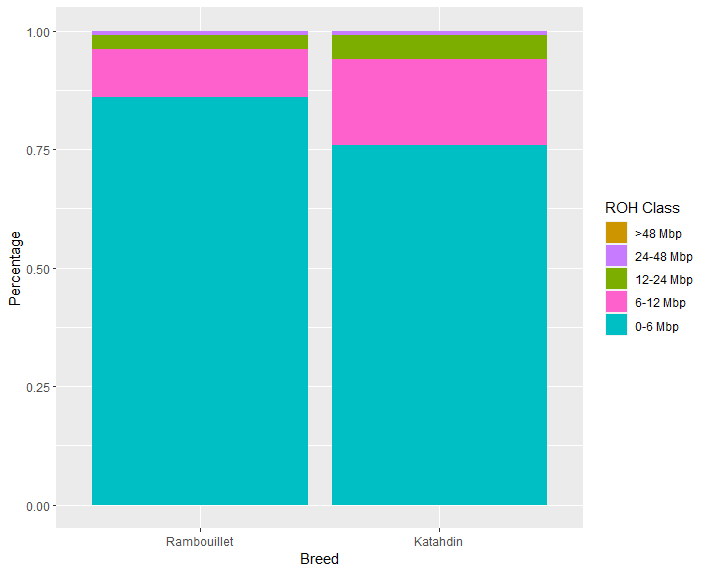

Supplement: Supplementary file 4 — Additional file 4: Figure S2. Stacked bar plot for the proportion of ROH called within each ROH size class by breed. [file 12711_2024_905_MOESM4_ESM.png]

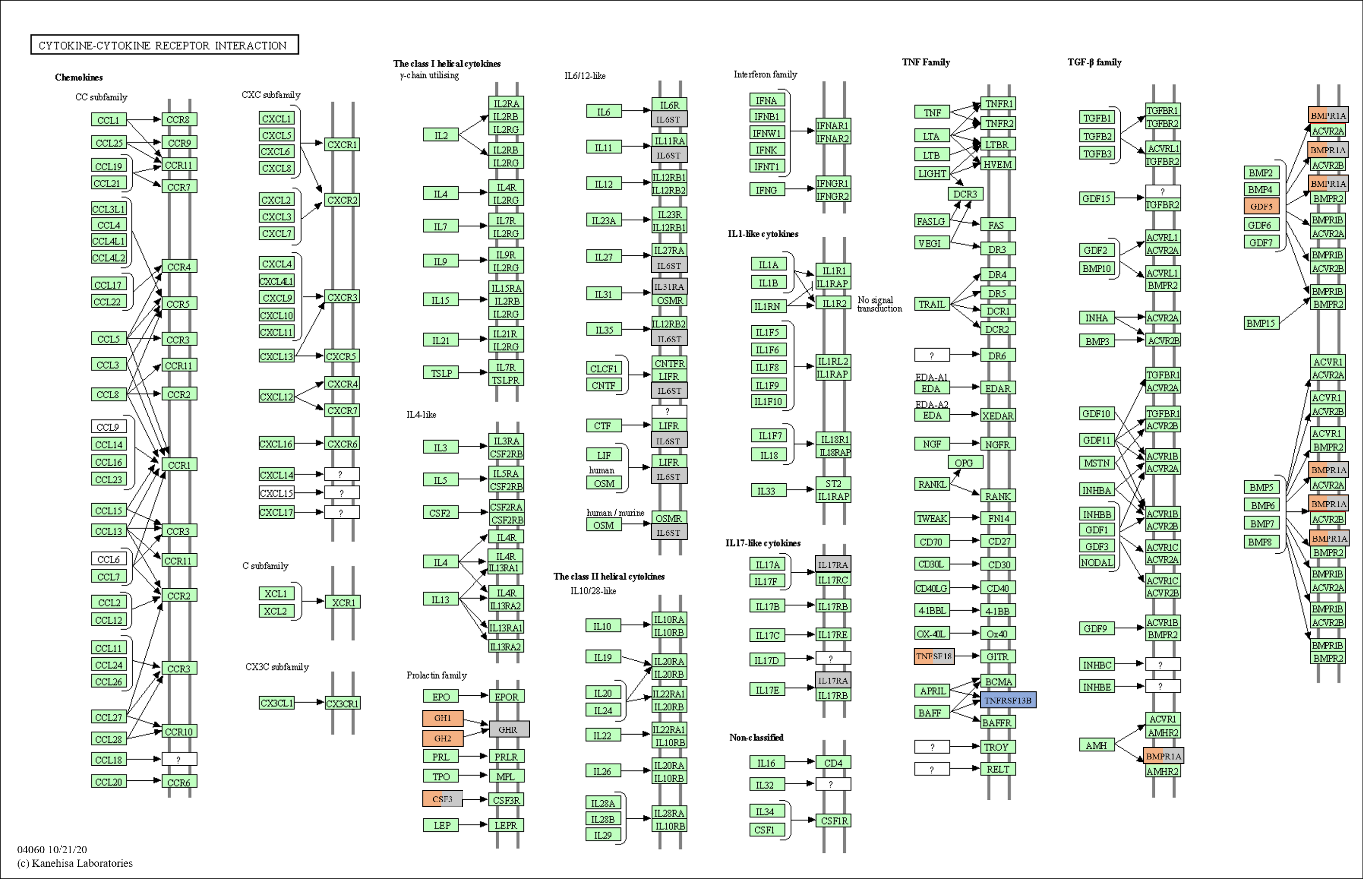

Supplement: Supplementary file 20 — Additional file 20: Figure S3. Genes belonging to cytokine-cytokine receptor interaction pathways identified from breed FST analyses. Genes identified in Katahdin-Dorper FST analysis are in blue, genes identified from the Katahdin-Rambouillet FST analysis are in orange, and genes identified from the Rambouillet-Dorper FST analysis are in gray. Pathway figure is modified from Kanehisa Laboratories (KEGG). [file 12711_2024_905_MOESM20_ESM.png]
