# Supplementary material for: Genetic diversity of United States Rambouillet, Katahdin and Dorper sheep
Source: Genet Sel Evol. 2024 Jul 30;56:56. doi: 10.1186/s12711-024-00905-7 (PMC11290166; doi:10.1186/s12711-024-00905-7)
Supplement: Supplementary file 2 — Additional file 2: Table S1. Effective population size (Ne) for Dorper sheep. The rate of change (m) between each pair of generations is reported, with the overall rate of change reported in italics. [file 12711_2024_905_MOESM2_ESM.docx]

| **Generations Ago** | ***N_e_*** | **Average Distance (bp)** | **Average LD (*r^2^*) ± SD** | **Number pairwise comparisons** | **Rate of Change (m)** |
| --- | --- | --- | --- | --- | --- |
| 13 | 95 | 3,749,343 | 0.0658 ± 0.09 | 122,677 | 2.06 |
| 15 | 101 | 3,273,360 | 0.0702 ± 0.10 | 111,039 | 3.00 |
| 17 | 109 | 2,844,335 | 0.0744 ± 0.10 | 100,713 | 4.00* |
| 20 | 118 | 2,460,309 | 0.0790 ± 0.11 | 89,873 | 3.00 |
| 23 | 129 | 2,116,788 | 0.0837 ± 0.11 | 79,977 | 3.67 |
| 27 | 142 | 1,811,164 | 0.0885 ± 0.12 | 71,357 | 3.25 |
| 32 | 156 | 1,540,844 | 0.0944 ± 0.12 | 63,557 | 2.80 |
| 38 | 174 | 1,303,367 | 0.0993 ± 0.13 | 55,836 | 3.00 |
| 45 | 197 | 1,095,408 | 0.1038 ± 0.13 | 49,128 | 3.29 |
| 54 | 222 | 914,143 | 0.1095 ± 0.14 | 42,769 | 2.78 |
| 65 | 258 | 757,862 | 0.1135 ± 0.14 | 36,849 | 3.27 |
| 80 | 297 | 623,727 | 0.1189 ± 0.15 | 31,675 | 2.60 |
| 98 | 345 | 509,526 | 0.1244 ± 0.16 | 27,113 | 2.67 |
| 120 | 407 | 413,586 | 0.1292 ± 0.16 | 22,585 | 2.82 |
| 149 | 495 | 333,645 | 0.1315 ± 0.16 | 18,758 | 3.03 |
| 187 | 584 | 267,154 | 0.1381 ± 0.17 | 15,428 | 2.34 |
| 234 | 711 | 213,251 | 0.1416 ± 0.17 | 12,586 | 2.70 |
| 293 | 886 | 170,164 | 0.1423 ± 0.17 | 10,001 | 2.97 |
| 367 | 996 | 136,134 | 0.1557 ± 0.19 | 7732 | 1.49 |
| 454 | 1151 | 110,011 | 0.1648 ± 0.20 | 5879 | 1.78 |
| 553 | 1373 | 90,369 | 0.1677 ± 0.20 | 4471 | 2.24 |
| 657 | 1504 | 76,031 | 0.1794 ± 0.22 | 3066 | 1.26 |
| 758 | 1671 | 65,903 | 0.1850 ± 0.22 | 2207 | 1.65 |
| 846 | 1756 | 59,081 | 0.1942 ± 0.23 | 1524 | 0.97 |
| 912 | 1891 | 54,775 | 0.1944 ± 0.23 | 923 | *2.05* |
