# Supplementary material for: Genetic diversity of United States Rambouillet, Katahdin and Dorper sheep
Source: Genet Sel Evol. 2024 Jul 30;56:56. doi: 10.1186/s12711-024-00905-7 (PMC11290166; doi:10.1186/s12711-024-00905-7)
Supplement: Supplementary file 3 — Additional file 3: Table S2. Percentage of ROH and mean size of ROH by breed and by Mbp class. [file 12711_2024_905_MOESM3_ESM.docx]

|  | **0-6 Mbp** | **6-12 Mbp** | **12-24 Mbp** | **24-48 Mbp** | **>48 Mbp** |
| --- | --- | --- | --- | --- | --- |
| **Percent ROH Rambouillet** | 0.86 | 0.10 | 0.03 | 0.01 | 0.00 |
| **Percent ROH Katahdin** | 0.75 | 0.18 | 0.05 | 0.01 | 0.00 |
| **Mean ROH Size Rambouillet** | 3.01 | 8.08 | 16.24 | 31.49 | 60.19 |
| **Mean ROH Size Katahdin** | 3.19 | 8.21 | 15.92 | 31.20 | 58.65 |
