# Supplementary material for: Genetic diversity of United States Rambouillet, Katahdin and Dorper sheep
Source: Genet Sel Evol. 2024 Jul 30;56:56. doi: 10.1186/s12711-024-00905-7 (PMC11290166; doi:10.1186/s12711-024-00905-7)
Supplement: Supplementary file 5 — Additional file 5: Table S3. ROH islands identified in Dorper sheep. ROH islands are defined by SNPs called within a ROH for more than 50% of Dorper sheep. *indicates these regions have overlap between Dorper and Rambouillet ROH calls. [file 12711_2024_905_MOESM5_ESM.docx]

| **Chr** | **Number of SNPs** | **AVG % SNP in Run** | **Max % SNP in Run** | **Range (bp)** |
| --- | --- | --- | --- | --- |
| 3 | 70 | 54.64 | 58.87 | 118,280,425 - 139,787,870* |
| 4 | 13 | 52.89 | 53.58 | 34,788,840 - 35,608,864 |
| 5 | 21 | 51.30 | 54.34 | 11,422,188 - 12,842,088 |
| 6 | 43 | 58.87 | 67.55 | 37,344,980 - 39,920,324* |
| 7 | 38 | 51.29 | 52.08 | 34,659,765 - 36,624,406 |
| 9 | 32 | 55.20 | 55.85 | 5,716,527 - 8,618,943 |
| 10 | 98 | 54.60 | 60.00 | 27,101,954 - 39,603,890 |
| 12 | 9 | 50.65 | 50.94 | 52,652,775 - 53,370,969 |
| 13 | 35 | 59.25 | 62.26 | 54,757,695 - 56,723,175 |
| 19 | 25 | 56.15 | 59.25 | 56,182,684 - 58,180,756 |
