# Supplementary material for: Genetic diversity of United States Rambouillet, Katahdin and Dorper sheep
Source: Genet Sel Evol. 2024 Jul 30;56:56. doi: 10.1186/s12711-024-00905-7 (PMC11290166; doi:10.1186/s12711-024-00905-7)
Supplement: Supplementary file 6 — Additional file 6: Table S4. Results of KEGG Mapper Pathway analysis of genes located within Katahdin ROH islands. [file 12711_2024_905_MOESM6_ESM.docx]

| **Katahdin ROH KEGG Mapper Pathways** | **Genes** |
| --- | --- |
| Adherens junction; Cell adhesion molecules | *PTPRM* |
| Alcoholism; Apelin signaling pathway; Chemokine signaling pathway; Cholinergic synapse; Circadian entrainment; GABAergic synapse; Glutamatergic synapse; Human cytomegalovirus infection; Human immunodeficiency virus 1 infection; Kaposi sarcoma-associated herpesvirus infection; Morphine addiction; Relaxin signaling pathway; Serotonergic synapse | *GNG4* |
| Aldosterone synthesis and secretion; cAMP signaling pathway; Cortisol synthesis and secretion; Cushing syndrome | *MC2R* |
| Alzheimer disease; Huntington disease; Pathways of neurodegeneration; Prion disease | *NDUFV2, TUBB6* |
| Amoebiasis | *GNAL, LAMA1* |
| AMPK signaling pathway | *CIDEA* |
| Amyotrophic lateral sclerosis | *HNRNPA2B1, NDUFV2, SEH1L, TUBB6* |
| Arrhythmogenic right ventricular cardiomyopathy; Dilated cardiomyopathy; ECM-receptor interaction; Focal adhesion; Human papillomavirus infection; Hypertrophic cardiomyopathy; Small cell lung cancer; Toxoplasmosis; Viral myocarditis | *LAMA1* |
| Calcium signaling pathway; Chagas disease; Olfactory transduction | *GNAL* |
| Chemical carcinogenesis - reactive oxygen species; Diabetic cardiomyopathy; Non-alcoholic fatty liver disease; Oxidative phosphorylation; Thermogenesis | *NDUFV2* |
| Cholesterol metabolism | *VAPA* |
| Dopaminergic synapse | *GNAL, GNG4* |
| Endocytosis | *RAB31, CHMP1B* |
| Gap junction; Pathogenic Escherichia coli infection; Phagosome; Salmonella infection | *TUBB6* |
| Glycosylphosphatidylinositol (GPI)-anchor biosynthesis | *MPPE1* |
| Inositol phosphate metabolism; Phosphatidylinositol signaling system | *IMPA2* |
| JAK-STAT signaling pathway | *PTPN2* |
| Mannose type O-glycan biosynthesis | *B3GALNT2* |
| Metabolic pathways | *B3GALNT2, IMPA2, NDUFV2, MPPE1, GGPS1* |
| mRNA surveillance pathway | *RNMT* |
| mTOR signaling pathway; Nucleocytoplasmic transport | *SEH1L* |
| Necroptosis | *CHMP1B* |
| Neuroactive ligand-receptor interaction | *MC2R, MC5R* |
| Pancreatic cancer | *RALBP1* |
| Parkinson disease | *GNAL, NDUFV2, TUBB6* |
| Pathways in cancer | *RALBP1, GNG4, LAMA1* |
| PI3K-Akt signaling pathway | *GNG4, LAMA1* |
| Ras signaling pathway | *RALBP1, GNG4* |
| Retrograde endocannabinoid signaling | *GNG4, NDUFV2* |
| Spinocerebellar ataxia | *AFG3L2* |
| Terpenoid backbone biosynthesis | *GGPS1* |
| Wnt signaling pathway | *APCDD1* |
