# Supplementary material for: Genetic diversity of United States Rambouillet, Katahdin and Dorper sheep
Source: Genet Sel Evol. 2024 Jul 30;56:56. doi: 10.1186/s12711-024-00905-7 (PMC11290166; doi:10.1186/s12711-024-00905-7)
Supplement: Supplementary file 7 — Additional file 7: Table S5. Results of KEGG Mapper Pathway analysis of genes located within Rambouillet ROH islands. [file 12711_2024_905_MOESM7_ESM.docx]

| **Rambouillet ROH KEGG Mapper Pathways** | **Genes** |
| --- | --- |
| ABC transporters | *ABCD2, ABCG2* |
| Adipocytokine signaling pathway; AMPK signaling pathway; Glucagon signaling pathway; Insulin resistance; Longevity regulating pathway | *PPARGC1A* |
| Adrenergic signaling in cardiomyocytes; cAMP signaling pathway; Leukocyte transendothelial migration; Long-term potentiation; Phospholipase D signaling pathway; Rap1 signaling pathway; Serotonergic synapse | *RAPGEF3* |
| Alcoholic liver disease | *PPARGC1A, IRAK4* |
| Alcoholism; Neutrophil extracellular trap formation | *HDAC7* |
| Alzheimer disease | *ATP5F1A, SNCA* |
| Aminoacyl-tRNA biosynthesis; Selenocompound metabolism | *SEPSECS* |
| Amyotrophic lateral sclerosis | *HNRNPA1, ATP5F1A* |
| Antifolate resistance | *DHFR, ABCG2* |
| Apelin signaling pathway | *PPARGC1A, RPS6, SPP1* |
| Arginine and proline metabolism; Glutathione metabolism | *LAP3* |
| Axon guidance | *SLIT2* |
| Bacterial invasion of epithelial cells; Fc gamma R-mediated phagocytosis; Regulation of actin cytoskeleton; Tight junction | *ARPC3* |
| Bile secretion | *ABCG2* |
| Biosynthesis of amino acids; Carbon metabolism; Fructose and mannose metabolism; Glycolysis / Gluconeogenesis | *TPI1* |
| Biosynthesis of cofactors; Folate biosynthesis; One carbon pool by folate | *DHFR* |
| Calcium signaling pathway | *STIM2, CCKAR* |
| Cell adhesion molecules | *CNTN1* |
| Cell cycle; Oocyte meiosis | *ANAPC4, RBX1* |
| Chagas disease; Hepatitis B; Herpes simplex virus 1 infection; Influenza A; Leishmaniasis; Lipid and atherosclerosis; Measles; Neurotrophin signaling pathway; NF-kappa B signaling pathway; NOD-like receptor signaling pathway; Pertussis; Toxoplasmosis | *IRAK4* |
| Chemical carcinogenesis - reactive oxygen species; Diabetic cardiomyopathy; Oxidative phosphorylation; Prion disease | *ATP5F1A* |
| Chemical carcinogenesis - receptor activation; Endocrine and other factor-regulated calcium reabsorption | *VDR* |
| Choline metabolism in cancer; Glycerophospholipid metabolism | *LYPLA1* |
| Circadian rhythm; Nucleotide excision repair; Pathways in cancer; Protein processing in endoplasmic reticulum; Renal cell carcinoma; TGF-beta signaling pathway | *RBX1* |
| Coronavirus disease - COVID19 | *RPL10A, IRAK4, RPL37, RPS6* |
| ECM-receptor interaction; Focal adhesion | *COL2A1, IBSP, SPP1* |
| EGFR tyrosine kinase inhibitor resistance; mTOR signaling pathway; Proteoglycans in cancer | *RPS6* |
| Endocytosis | *ARPC3, SNX4* |
| Epstein-Barr virus infection | *RBPJ, IRAK4* |
| GABAergic synapse; Glutamatergic synapse | *SLC38A2, SLC38A1* |
| Glycosylphosphatidylinositol (GPI)-anchor biosynthesis | *PIGY* |
| GnRH secretion | *SPP1* |
| Hepatocellular carcinoma | *ARID2* |
| HIF-1 signaling pathway | *RPS6, RBX1* |
| Human immunodeficiency virus 1 infection | *IRAK4, RBX1* |
| Human papillomavirus infection; PI3K-Akt signaling pathway | *COL2A1, IBSP, RBPJ, SPP1* |
| Human T-cell leukemia virus 1 infection | *ANAPC4, RAN* |
| Huntington disease | *PPARGC1A, ATP5F1A* |
| Inositol phosphate metabolism | *PI4K2B, TPI1* |
| Insulin secretion; Pancreatic secretion | *CCKAR* |
| Insulin signaling pathway | *PPARGC1A, RPS6* |
| Long-term depression | *GRID2* |
| MAPK signaling pathway | *IRAK4, PTPRR* |
| Metabolic pathways | *DHFR, ATP5F1A, LAP3, SEPSECS, PI4K2B, GBA3, TPI1, PIGY* |
| Mineral absorption; Parathyroid hormone synthesis, secretion and action | *SLC34A2, VDR* |
| Neuroactive ligand-receptor interaction | *GRID2, CCKAR* |
| Notch signaling pathway; Spinocerebellar ataxia; Th1 and Th2 cell differentiation | *RBPJ* |
| Nucleocytoplasmic transport; Ribosome biogenesis in eukaryotes; Viral life cycle - HIV-1 | *RAN* |
| Other types of O-glycan biosynthesis | *GXYLT1* |
| Parkinson disease; Pathways of neurodegeneration | *LRRK2, ATP5F1A, SNCA* |
| Pathogenic Escherichia coli infection; Salmonella infection; Yersinia infection | *ARPC3, IRAK4* |
| Peroxisome | *ABCD2* |
| Phosphatidylinositol signaling system | *PI4K2B* |
| Progesterone-mediated oocyte maturation | *ANAPC4* |
| Protein digestion and absorption | *COL2A1, SLC38A2* |
| Ribosome | *RPL10A, RPL37, RPS6* |
| Shigellosis | *ARPC3, RBX1* |
| Spliceosome | *DHX15, HNRNPA1, RBM17* |
| Starch and sucrose metabolism | *GBA3* |
| Thermogenesis | *PPARGC1A, ATP5F1A, RPS6* |
| Toll-like receptor signaling pathway | *IRAK4, SPP1* |
| Tuberculosis | *IRAK4, VDR* |
| Ubiquitin mediated proteolysis | *ANAPC4, HERC3, RBX1* |
| Viral carcinogenesis | *RBPJ, HDAC7* |
| Wnt signaling pathway | *PRICKLE1, RBX1* |
