# Supplementary material for: Genetic diversity of United States Rambouillet, Katahdin and Dorper sheep
Source: Genet Sel Evol. 2024 Jul 30;56:56. doi: 10.1186/s12711-024-00905-7 (PMC11290166; doi:10.1186/s12711-024-00905-7)
Supplement: Supplementary file 9 — Additional file 9: Table S7. Summary of FST regions by chromosome and breed comparison. [file 12711_2024_905_MOESM9_ESM.docx]

|  | **Katahdin-Dorper** | | | **Rambouillet-Dorper** | | | **Katahdin-Rambouillet** | | |
| --- | --- | --- | --- | --- | --- | --- | --- | --- | --- |
| **Chr** | **SNP Count** | **Number of Regions** | **Average Length of Region** | **SNP Count** | **Number of Regions** | **Average Length of Region** | **SNP Count** | **Number of Regions** | **Average Length of Region** |
| 1 | 2 | 1 | 216,990.00 |  |  |  | 8 | 4 | 255,337.00 |
| 2 | 6 | 3 | 241,687.67 | 10 | 5 | 234,850.00 | 2 | 1 | 207,181.00 |
| 3 | 33 | 14 | 314,846.57 | 24 | 9 | 389,556.78 | 27 | 11 | 289,008.91 |
| 4 | 8 | 4 | 247,705.25 | 2 | 1 | 246,060.00 | 9 | 3 | 341,430.67 |
| 5 |  |  |  | 4 | 2 | 273,374.50 | 4 | 2 | 234,115.50 |
| 6 | 4 | 2 | 230,169.50 | 39 | 15 | 355,158.00 | 28 | 10 | 382,793.80 |
| 7 | 2 | 1 | 286,010.00 | 4 | 2 | 300,910.00 | 4 | 2 | 237,645.00 |
| 8 | 10 | 4 | 282,549.50 | 4 | 2 | 223,162.50 | 2 | 1 | 209,858.00 |
| 9 | 2 | 1 | 267,295.00 | 6 | 3 | 287,341.33 |  |  |  |
| 10 | 11 | 4 | 357,096.75 | 11 | 5 | 274,372.20 | 9 | 3 | 333,838.33 |
| 11 | 4 | 2 | 293,762.00 | 9 | 4 | 266,221.25 | 15 | 6 | 279,958.50 |
| 12 | 4 | 2 | 226,448.50 | 2 | 1 | 238,214.00 | 2 | 1 | 274,558.00 |
| 13 | 2 | 1 | 236,274.00 | 6 | 3 | 269,164.33 | 8 | 3 | 270,602.67 |
| 14 | 2 | 1 | 254,025.00 |  |  |  |  |  |  |
| 15 |  |  |  | 11 | 5 | 308,026.40 | 2 | 1 | 252,791.00 |
| 16 |  |  |  | 10 | 5 | 282,648.80 | 9 | 3 | 322,504.33 |
| 17 |  |  |  | 3 | 1 | 346,635.00 | 2 | 1 | 220,537.00 |
| 18 |  |  |  | 5 | 2 | 370,241.00 |  |  |  |
| 19 | 2 | 1 | 248,673.00 | 4 | 2 | 243,718.00 | 5 | 2 | 290,365.00 |
| 20 |  |  |  | 3 | 1 | 340,868.00 |  |  |  |
| 21 | 4 | 2 | 234,874.50 |  |  |  |  |  |  |
| 22 | 2 | 1 | 292,801.00 | 2 | 1 | 216,911.00 |  |  |  |
| 23 | 8 | 4 | 280,955.25 | 4 | 2 | 265,424.00 | 7 | 3 | 312,922.33 |
| 24 |  |  |  |  |  |  |  |  |  |
| 25 | 3 | 1 | 311,560.00 | 4 | 2 | 282,501.50 | 14 | 6 | 311,021.17 |
| 26 |  |  |  |  |  |  |  |  |  |
| Total | 109 | 49 | 4,823,723.49 | 167 | 73 | 6,015,358.59 | 157 | 63 | 5,026,468.21 |
