# Supplementary material for: Genetic diversity of United States Rambouillet, Katahdin and Dorper sheep
Source: Genet Sel Evol. 2024 Jul 30;56:56. doi: 10.1186/s12711-024-00905-7 (PMC11290166; doi:10.1186/s12711-024-00905-7)
Supplement: Supplementary file 10 — Additional file 10: Table S8. List of SNPs identified from both Katahdin-Rambouillet FST analysis and Katahdin or Rambouillet ROH islands. [file 12711_2024_905_MOESM10_ESM.docx]

| SNP | CHR | BP |
| --- | --- | --- |
| OAR3_148764019.1 | 3 | 139,191,812 |
| OAR3_148886217.1 | 3 | 139,307,125 |
| OAR3_156691225.1 | 3 | 147,094,688 |
| OAR3_156734590.1 | 3 | 147,144,924 |
| OAR6_37483582.1 | 6 | 34,174,998 |
| OAR6_37533580.1 | 6 | 34,225,519 |
| OAR6_37546315.1 | 6 | 34,241,051 |
| OAR6_37625163.1 | 6 | 34,315,218 |
| OAR6_37669211.1 | 6 | 34,370,580 |
| OAR6_37861888.1 | 6 | 34,566,514 |
| s26384.1 | 6 | 34,737,092 |
| OAR6_42484920_X.1 | 6 | 38,927,593 |
| OAR6_42557643.1 | 6 | 39,000,766 |
| OAR6_42834740.1 | 6 | 39,195,269 |
| OAR6_45273992.1 | 6 | 41,295,843 |
| OAR6_45438896.1 | 6 | 41,457,440 |
| s51718.1 | 6 | 42,809,260 |
| s55289.1 | 6 | 47,317,319 |
| OAR23_46373969.1 | 23 | 43,975,354 |
| OAR23_46850769.1 | 23 | 44,460,728 |
| OAR23_46900744.1 | 23 | 44,502,153 |
| OAR23_46996994.1 | 23 | 44,599,758 |
| s44881.1 | 25 | 6,938,538 |
| s09536.1 | 25 | 7,667,075 |
| OAR25_8218556.1 | 25 | 7,728,170 |
