# Supplementary material for: Genetic diversity of United States Rambouillet, Katahdin and Dorper sheep
Source: Genet Sel Evol. 2024 Jul 30;56:56. doi: 10.1186/s12711-024-00905-7 (PMC11290166; doi:10.1186/s12711-024-00905-7)
Supplement: Supplementary file 11 — Additional file 11: Table S9. Results of GO enrichment analysis of genes located within Katahdin-Rambouillet FST regions. [file 12711_2024_905_MOESM11_ESM.docx]

| **GO Results of Katahdin-Rambouillet F_ST_** | **Reference #** | **Observed #** | **Expected** | **Fold Enrichment** | **+/-** | **FDR** |
| --- | --- | --- | --- | --- | --- | --- |
| **Biological Process** |  |  |  |  |  |  |
| Negative regulation of cellular glucuronidation | 8 | 5 | 0.09 | 56.2 | + | 2.83E-03 |
| Regulation of cellular glucuronidation | 9 | 5 | 0.1 | 49.96 | + | 1.09E-03 |
| Negative regulation of glucuronosyltransferase activity | 8 | 5 | 0.09 | 56.2 | + | 1.42E-03 |
| Regulation of glucuronosyltransferase activity | 8 | 5 | 0.09 | 56.2 | + | 9.44E-04 |
| Cellular glucuronidation | 20 | 5 | 0.22 | 22.48 | + | 2.10E-02 |
| Glucuronate metabolic process | 25 | 5 | 0.28 | 17.98 | + | 3.85E-02 |
| Uronic acid metabolic process | 25 | 5 | 0.28 | 17.98 | + | 4.49E-02 |
| **Molecular Function** |  |  |  |  |  |  |
| UDP-glycosyltransferase activity | 146 | 11 | 1.62 | 6.77 | + | 6.96E-03 |
