# Supplementary material for: Genetic diversity of United States Rambouillet, Katahdin and Dorper sheep
Source: Genet Sel Evol. 2024 Jul 30;56:56. doi: 10.1186/s12711-024-00905-7 (PMC11290166; doi:10.1186/s12711-024-00905-7)
Supplement: Supplementary file 12 — Additional file 12: Table S10. KEGG Mapper pathway results for query of Katahdin-Rambouillet genes against the Homo sapiens reference database. [file 12711_2024_905_MOESM12_ESM.docx]

| **Katahdin-Rambouillet F_ST_ KEGG Mapper Pathway** | **Gene** |
| --- | --- |
| Adherens junction; Tight junction; Leukocyte transendothelial migration | *AFDN* |
| Alcoholic liver disease | *SREBF1, MAP3K14* |
| Aldosterone synthesis and secretion ; Cortisol synthesis and secretion; Cushing syndrome | *ADCY6* |
| Alzheimer disease | *SLC39A9, PSMC5, PSMD3, SDHA* |
| Amino sugar and nucleotide sugar metabolism | *HEXA, NAGK, UAP1* |
| AMPK signaling pathway | *RPTOR, SREBF1, STRADA* |
| Amyotrophic lateral sclerosis | *PSMC5, PSMD3, BCL2L1, SDHA* |
| Apelin signaling pathway; Chemokine signaling pathway; Cholinergic synapse; Retrograde endocannabinoid signaling ; Circadian entrainment; Morphine addiction | *ADCY6, GNG4* |
| Apoptosis | *NTRK1, BCL2L1, MAP3K14* |
| Apoptosis - multiple species | *BIRC6, BCL2L1* |
| Autophagy - animal | *RPTOR, BCL2L1* |
| Autophagy - other; MicroRNAs in cancer | *RPTOR* |
| Axon guidance | *RND1* |
| B cell receptor signaling pathway | *CD79B* |
| Bile secretion | *ADCY6, UGT1A6, UGT1A9, UGT1A4, UGT1A1, UGT1A3, SLC4A5, SLC9A3* |
| Biosynthesis of cofactors | *MTHFD2, UGT1A6, UGT1A9, UGT1A4, UGT1A1, UGT1A3* |
| Biosynthesis of nucleotide sugars | *NAGK, UAP1* |
| Calcium signaling pathway; Central carbon metabolism in cancer | *NTRK1* |
| cAMP signaling pathway | *ADCY6, AFDN* |
| Carbon metabolism; Citrate cycle (TCA cycle) ; Diabetic cardiomyopathy | *SDHA* |
| Cardiac muscle contraction; Hypertrophic cardiomyopathy; Arrhythmogenic right ventricular cardiomyopathy | *CACNB3* |
| cGMP-PKG signaling pathway; Gap junction; Insulin secretion; Regulation of lipolysis in adipocytes; Ovarian steroidogenesis; Estrogen signaling pathway; Thyroid hormone synthesis; Melanogenesis; Renin secretion; Salivary secretion; Gastric acid secretion; Pancreatic secretion; Endocrine and other factor-regulated calcium reabsorption; Taste transduction; Endocrine resistance | *ADCY6* |
| Chemical carcinogenesis - reactive oxygen species | *SDHA, MAP3K14* |
| Chemical carcinogenesis - receptor activation | *ADCY6, UGT1A6, UGT1A9, UGT1A4, UGT1A1, UGT1A3* |
| Circadian rhythm | *NR1D1* |
| Collecting duct acid secretion; Human papillomavirus infection; Vibrio cholerae infection; Rheumatoid arthritis | *ATP6V1B1* |
| Complement and coagulation cascades | *PROCR, F2RL3* |
| Coronavirus disease - COVID-19 | *CSF3, RPL10A, RPL5, RPL6, RPL7, RPL23A, RPL37* |
| Cytokine-cytokine receptor interaction | *CSF3, GH1, BMPR1A, GDF5, TNFSF18* |
| Dopaminergic synapse; Serotonergic synapse; Kaposi sarcoma-associated herpesvirus infection; Alcoholism | *GNG4* |
| EGFR tyrosine kinase inhibitor resistance | *EIF4E, NF1, BCL2L1* |
| Endocytosis | *RAB11FIP4* |
| Epithelial cell signaling in Helicobacter pylori infection | *ATP6V1B1, MAP3K14* |
| Epstein-Barr virus infection | *PSMC5, PSMD3, MAP3K14* |
| Ferroptosi | *PCBP1, NCOA4* |
| GABAergic synapse | *ADCY6, GNG4, SLC38A2, SLC38A1* |
| Galactose metabolism | *LALBA* |
| Glutamatergic synapse | *ADCY6, GNG4, SLC38A2, SLC1A3, SLC38A1* |
| Glycerolipid metabolism | *GPAT3* |
| Glycerophospholipid metabolism | *GPAT3* |
| GnRH signaling pathway | *ADCY6, MAP3K3* |
| Growth hormone synthesis, secretion and action | *ADCY6, GH1, SOCS2* |
| Hematopoietic cell lineage; IL-17 signaling pathway; Malaria | *CSF3* |
| Hepatocellular carcinoma | *BCL2L1, SMARCD2* |
| Herpes simplex virus 1 infection | *BCL2L1* |
| HIF-1 signaling pathway | *EIF4E* |
| Hippo signaling pathway | *MOB1A, BMPR1A, GDF5, DLG5* |
| Hippo signaling pathway - multiple species | *MOB1A* |
| Homologous recombination | *ABRAXAS1* |
| Human cytomegalovirus infection | *ADCY6, GNG4, ARHGEF11* |
| Human immunodeficiency virus 1 infection | *GNG4, BCL2L1* |
| Human T-cell leukemia virus 1 infection | *ADCY6, MAP3K3, BCL2L1, MAP3K14* |
| Huntington disease | *PSMC5, PSMD3, SDHA, SLC1A3* |
| Inflammatory mediator regulation of TRP channels | *ADCY6, NTRK1, TRPM8* |
| Inositol phosphate metabolism; Phosphatidylinositol signaling system | *INPP4A* |
| Insulin resistance | *SLC27A6, SREBF1* |
| Insulin signaling pathway | *EIF4E, PRKAR2B, RPTOR, SREBF1, SOCS2* |
| JAK-STAT signaling pathway | *CSF3, GH1, BCL2L1, SOCS2* |
| Legionellosis; Leishmaniasis | *EEF1A1* |
| Longevity regulating pathway | *ADCY6, EIF4E, RPTOR* |
| Longevity regulating pathway - multiple species | *ADCY6, RPTOR* |
| Lysine degradation | *SETMAR* |
| Lysosome | *SUMF1, HEXA* |
| Mannose type O-glycan biosynthesis | *B3GALNT2* |
| MAPK signaling pathway | *MAP3K3, NF1, NTRK1, CACNB3, MAP3K14* |
| Metabolic pathways | *MTHFD2, ADCY6, MGAT4A, B3GALNT2, HEXA, INPP4A, LALBA, ATP6V1B1, UGT1A6, UGT1A9, UGT1A4, UGT1A1, UGT1A3, NAGK, GALNT16, GBA3, SDHA, SETMAR, UAP1, GPAT3* |
| Metabolism of xenobiotics by cytochrome P450 | *UGT1A6, UGT1A9, UGT1A4, UGT1A1, UGT1A3* |
| Mineral absorption | *SLC9A3* |
| Mitophagy - animal; p53 signaling pathway; NOD-like receptor signaling pathway; Pancreatic cancer; Chronic myeloid leukemia ; Small cell lung cancer ; Measles ; Toxoplasmosis ; Lipid and atherosclerosis; Platinum drug resistance | *BCL2L1* |
| mRNA surveillance pathway | *FIP1L1* |
| mTOR signaling pathway | *EIF4E, ATP6V1B1, RPTOR, STRADA* |
| Mucin type O-glycan biosynthesis | *GALNT16* |
| Neuroactive ligand-receptor interaction | *RXFP2, GH1, THRA, F2RL3* |
| Neurotrophin signaling pathway | *MAP3K3, NTRK1* |
| NF-kappa B signaling pathway | *BCL2L1, MAP3K14* |
| N-Glycan biosynthesis | *MGAT4A* |
| Non-alcoholic fatty liver disease | *SDHA, SREBF1* |
| Nucleocytoplasmic transport | *EEF1A1* |
| Olfactory transduction | *OR2D3, OR10A5, OR2AG1, OR10A2, OR8S1* |
| One carbon pool by folate | *MTHFD2* |
| Oocyte meiosis; Progesterone-mediated oocyte maturation | *ADCY6, CPEB2* |
| Other types of O-glycan biosynthesis | *B3GLCT, GALNT16* |
| Oxidative phosphorylation | *ATP6V1B1, SDHA* |
| Oxytocin signaling pathway; Adrenergic signaling in cardiomyocytes; Dilated cardiomyopathy | *ADCY6, CACNB3* |
| Parathyroid hormone synthesis, secretion and action | *MMP24, ADCY6, ARHGEF11* |
| Parkinson disease | *LRRK2, SLC39A9, PSMC5, PSMD3, BCL2L1, SDHA* |
| Pathogenic Escherichia coli infection | *ARHGEF11* |
| Pathways in cancer | *ADCY6, GNG4, NTRK1, BCL2L1, NCOA4, F2RL3, ARHGEF11* |
| Pathways of neurodegeneration - multiple diseases | *LRRK2, PSMC5, PSMD3, BCL2L1, SDHA* |
| PD-L1 expression and PD-1 checkpoint pathway in cancer | *MAP3K3* |
| Pentose and glucuronate interconversions ; Ascorbate and aldarate metabolism ; Steroid hormone biosynthesis; Retinol metabolism ; Porphyrin metabolism; Drug metabolism - cytochrome P450 ; Drug metabolism - other enzymes ; Chemical carcinogenesis - DNA adducts | *UGT1A6, UGT1A9, UGT1A4, UGT1A1, UGT1A3* |
| Phagosome | *ATP6V1B1* |
| Phospholipase D signaling pathway | *ADCY6* |
| PI3K-Akt signaling pathway | *CSF3, EIF4E, GH1, GNG4, NTRK1, RPTOR, BCL2L1* |
| Platelet activation | *ADCY6, F2RL3* |
| PPAR signaling pathway | *SLC27A6* |
| Prion disease | *PSMC5, PSMD3, SDHA* |
| Prolactin signaling pathway; Type II diabetes mellitus | *SOCS2* |
| Proteasome; Spinocerebellar ataxia | *PSMC5, PSMD3* |
| Protein digestion and absorption | *SLC38A2, SLC9A3* |
| Protein processing in endoplasmic reticulum | *EDEM2* |
| Proximal tubule bicarbonate reclamation | *SLC9A3* |
| Purine metabolism | *ADCY6* |
| Rap1 signaling pathway | *ADCY6, AFDN, SIPA1L2, F2RL3* |
| Ras signaling pathway | *GNG4, AFDN, NF1, NTRK1, BCL2L1* |
| Regulation of actin cytoskeleton; Prostate cancer | *INSRR* |
| Relaxin signaling pathway | *ADCY6, RXFP2, GNG4* |
| Ribosome | *RPL10A, MRPS18C, RPL5, RPL6, RPL7, RPL23A, RPL37* |
| Ribosome biogenesis in eukaryotes | *EIF6* |
| RNA polymerase; Cytosolic DNA-sensing pathway | *POLR3A* |
| Salmonella infection | *PLEKHM1* |
| Shigellosis | *RPTOR, BCL2L1, UBE2N* |
| Signaling pathways regulating pluripotency of stem cells; Fluid shear stress and atherosclerosis | *BMPR1A* |
| Sphingolipid metabolism; Glycosaminoglycan degradation; Glycosphingolipid biosynthesis - globo and isoglobo series; Glycosphingolipid biosynthesis - ganglio series; Other glycan degradation | *HEXA* |
| Spliceosome | *SNRNP27, DDX42, PCBP1, DDX23* |
| Staphylococcus aureus infection | *DSG1* |
| Starch and sucrose metabolism | *GBA3* |
| Synaptic vesicle cycle | *ATP6V1B1, SLC1A3* |
| TGF-beta signaling pathway | *BMPR1A, GDF5* |
| Thermogenesis | *ADCY6, NDUFAF6, COA5, RPTOR, SDHA, SMARCD2* |
| Thyroid cancer | *NTRK1, NCOA4* |
| Thyroid hormone signaling pathway | *THRA, MED24* |
| TNF signaling pathway; C-type lectin receptor signaling pathway; T cell receptor signaling pathway; Intestinal immune network for IgA production; Osteoclast differentiation | *MAP3K14* |
| Transcriptional misregulation in cancer | *NTRK1, BCL2L1, CCNT1* |
| Ubiquitin mediated proteolysis | *BIRC6, UBE2N* |
| Various types of N-glycan biosynthesis | *MGAT4A, HEXA* |
| Vascular smooth muscle contraction | *ADCY6, ARHGEF11* |
| Vasopressin-regulated water reabsorption | *ADCY6* |
| Viral life cycle - HIV-1 | *CCNT1* |
