# Supplementary material for: Genetic diversity of United States Rambouillet, Katahdin and Dorper sheep
Source: Genet Sel Evol. 2024 Jul 30;56:56. doi: 10.1186/s12711-024-00905-7 (PMC11290166; doi:10.1186/s12711-024-00905-7)
Supplement: Supplementary file 13 — Additional file 13: Table S11. KEGG Mapper pathway results for query of Katahdin-Dorper genes against the Homo sapiens reference database. [file 12711_2024_905_MOESM13_ESM.docx]

| **Katahdin-Dorper F_ST_ Mapper Pathway** | **Genes** |
| --- | --- |
| Aldosterone-regulated sodium reabsorption; Thyroid hormone signaling pathway; Proximal tubule bicarbonate reclamation; Carbohydrate digestion and absorption | *ATP1B3* |
| Arrhythmogenic right ventricular cardiomyopathy; Hypertrophic cardiomyopathy | *ITGA7; CACNB3* |
| Autophagy - animal; Neutrophil extracellular trap formation | *ATG7; HMGB1* |
| Autophagy - other; Ferroptosis | *ATG7* |
| B cell receptor signaling pathway; Malaria; Hepatitis C | *CD81* |
| Bladder cancer; Leukocyte transendothelial migration | *MMP2* |
| Cortisol synthesis and secretion; GABAergic synapse; Parathyroid hormone synthesis, secretion and action; Renin secretion; Ovarian steroidogenesis; Melanogenesis; Longevity regulating pathway - multiple species; Retrograde endocannabinoid signaling; Longevity regulating pathway; Chemical carcinogenesis - receptor activation; Apelin signaling pathway; Vasopressin-regulated water reabsorption; Cushing syndrome | *ADCY6* |
| D-Amino acid metabolism; Peroxisome; Alanine, aspartate and glutamate metabolism | *DDO* |
| ECM-receptor interaction; PI3K-Akt signaling pathway; Human papillomavirus infection | *ITGA7; TNR* |
| Endocrine resistance; Estrogen signaling pathway | *ADCY6; MMP2* |
| Ether lipid metabolism; Glycerophospholipid metabolism | *LPCAT2* |
| Gastric acid secretion; Pancreatic secretion | *ADCY6; KCNQ1; ATP1B3* |
| Influenza A; Cytosolic DNA-sensing pathway; C-type lectin receptor signaling pathway | *NLRP3* |
| Legionellosis; Leishmaniasis | *EEF1A1* |
| Long-term potentiation; Nicotine addiction; Huntington disease; Alzheimer disease; Prion disease; Amphetamine addiction; Spinocerebellar ataxia; Systemic lupus erythematosus; Dopaminergic synapse; Alcoholism | *GRIN2B* |
| Lysosome; Starch and sucrose metabolism | *GAA* |
| Mismatch repair; DNA replication; Nucleotide excision repair | *RFC3* |
| Morphine addiction; Purine metabolism | *PDE10A; ADCY6* |
| Oocyte meiosis; Progesterone-mediated oocyte maturation | *ADCY6; CDC16* |
| PD-L1 expression and PD-1 checkpoint pathway in cancer; TNF signaling pathway; Toll-like receptor signaling pathway; Hepatitis B; Epstein-Barr virus infection; Toxoplasmosis; Alcoholic liver disease; Cellular senescence; Fc epsilon RI signaling pathway | *MAP2K3* |
| Phosphatidylinositol signaling system; Inositol phosphate metabolism | *INPP4A* |
| Primary immunodeficiency; Intestinal immune network for IgA production | *TNFRSF13B* |
| Regulation of lipolysis in adipocytes; Gap junction | *ADCY6; PRKG1* |
| Renal cell carcinoma; mTOR signaling pathway | *FLCN* |
| SNARE interactions in vesicular transport; Phagosome | *STX7* |
| Thyroid hormone synthesis; Insulin secretion; Bile secretion; Endocrine and other factor-regulated calcium reabsorption; Aldosterone synthesis and secretion | *ADCY6; ATP1B3* |
| Type II diabetes mellitus; Insulin signaling pathway; JAK-STAT signaling pathway; Prolactin signaling pathway | *SOCS2* |
| Various types of N-glycan biosynthesis; N-Glycan biosynthesis | *MGAT4A* |
| RNA degradation | *LSM5* |
| Spliceosome | *LSM5; RNU6-1; RNU1-1; CDC40; DDX23; EIF4A3* |
| Salivary secretion | *ADCY6; ATP1B3; PRKG1* |
| Human cytomegalovirus infection | *ADCY6* |
| Platelet activation | *ADCY6; PPP1R12A; PRKG1* |
| Pathways in cancer | *ADCY6; DCC; MMP2* |
| Glutamatergic synapse | *ADCY6; GRIN2B; GRM3* |
| Rap1 signaling pathway | *ADCY6; GRIN2B; MAP2K3* |
| Circadian entrainment | *ADCY6; GRIN2B; PRKG1* |
| Phospholipase D signaling pathway | *ADCY6; GRM3* |
| Inflammatory mediator regulation of TRP channels | *ADCY6; HRH1; MAP2K3* |
| Dilated cardiomyopathy | *ADCY6; ITGA7; CACNB3* |
| Cholinergic synapse | *ADCY6; KCNQ1* |
| Adrenergic signaling in cardiomyocytes | *ADCY6; KCNQ1; ATP1B3; CACNB3* |
| Growth hormone synthesis, secretion and action | *ADCY6; MAP2K3; SOCS2* |
| GnRH signaling pathway | *ADCY6; MMP2; MAP2K3* |
| cGMP-PKG signaling pathway | *ADCY6; PPP1R12A; ATP1B3; PRKG1* |
| Oxytocin signaling pathway | *ADCY6; PPP1R12A; CACNB3* |
| Vascular smooth muscle contraction | *ADCY6; PPP1R12A; PRKG1* |
| Thermogenesis | *ADCY6; PRKG1; MAP2K3* |
| Human T-cell leukemia virus 1 infection | *ADCY6; CDC16* |
| Relaxin signaling pathway | *ADCY6; RXFP2; MMP2* |
| Taste transduction | *ADCY6; TRPM5* |
| Fc gamma R-mediated phagocytosis | *CFL1* |
| Regulation of actin cytoskeleton | *CFL1; ITGA7; PPP1R12A* |
| Endocytosis | *DNAJC6* |
| Tight junction | *EPB41L4B* |
| Yersinia infection | *NLRP3; MAP2K3* |
| Pathogenic Escherichia coli infection | *NLRP3; MYO5B* |
| Shigellosis | *NLRP3; UBE2N* |
| Salmonella infection | *NLRP3; VPS41; MAP2K3* |
| Cardiac muscle contraction | *ATP1B3; CACNB3* |
| Mannose type O-glycan biosynthesis | *B3GALNT2* |
| Other types of O-glycan biosynthesis | *B3GLCT* |
| Circadian rhythm | *BHLHE41* |
| MAPK signaling pathway | *MAP2K3; CACNB3* |
| NF-kappa B signaling pathway | *CARD14* |
| Transcriptional misregulation in cancer | *CCNT1* |
| Cell cycle | *CDC16* |
| Drug metabolism - other enzymes | *CES1* |
| Axon guidance | *CFL1; DCC; RND1; SLIT2* |
| Human immunodeficiency virus 1 infection | *CFL1; MAP2K3* |
| Pertussis | *CFL1; NLRP3* |
| Olfactory transduction | *PRKG1* |
| Protein digestion and absorption | *KCNQ1; ATP1B3* |
| AGE-RAGE signaling pathway in diabetic complications | *MMP2* |
| Diabetic cardiomyopathy | *MMP2* |
| Amoebiasis | *SERPINB10; SERPINB4* |
| Chemokine signaling pathway | *ADCY6* |
| Cytokine-cytokine receptor interaction | *TNFRSF13B* |
| Colorectal cancer | *DCC* |
| Nucleocytoplasmic transport | *EEF1A1; UPF3A; EIF4A3* |
| Galactose metabolism | *GAA; LALBA* |
| Terpenoid backbone biosynthesis | *GGPS1* |
| Cocaine addiction | *GRIN2B; GRM3* |
| Pathways of neurodegeneration - multiple diseases | *GRIN2B; MAP2K3* |
| Amyotrophic lateral sclerosis | *GRIN2B; MAP2K3* |
| Porphyrin metabolism | *HEPHL1* |
| Mineral absorption | *HEPHL1; ATP1B3* |
| Base excision repair | *HMGB1* |
| Calcium signaling pathway | *HRH1* |
| Focal adhesion | *ITGA7; PPP1R12A; TNR* |
| Vibrio cholerae infection | *KCNQ1* |
| Proteoglycans in cancer | *MMP2; PPP1R12A* |
| Fluid shear stress and atherosclerosis | *MMP2* |
| Necroptosis | *NLRP3; HMGB1* |
| Lipid and atherosclerosis | *NLRP3; MAP2K3* |
| NOD-like receptor signaling pathway | *NLRP3; PANX1* |
| Coronavirus disease - COVID-19 | *NLRP3; RPL22; RPL27* |
| cAMP signaling pathway | *PDE10A; ADCY6; GRIN2B; PPP1R12A; ATP1B3* |
| Metabolic pathways | *PDE10A; ADCY6; MGAT4A; B3GALNT2; GAA; INPP4A; LALBA; LPCAT2; RDH5; DDO; GGPS1* |
| Renin-angiotensin system | *PREP* |
| Long-term depression | *PRKG1* |
| Viral life cycle - HIV-1 | *CPSF7; CCNT1* |
| Ras signaling pathway | *RASA3; GRIN2B* |
| Retinol metabolism | *RDH5* |
| Ribosome | *RPL22; RPL27* |
| Neuroactive ligand-receptor interaction | *RXFP2; GRIN2B; GRM3; HRH1* |
| Synaptic vesicle cycle | *SLC6A2* |
| MicroRNAs in cancer | *TNR* |
| Ubiquitin mediated proteolysis | *UBE2N; CDC16* |
| mRNA surveillance pathway | *UPF3A; CPSF7; EIF4A3* |
