# Supplementary material for: Genetic diversity of United States Rambouillet, Katahdin and Dorper sheep
Source: Genet Sel Evol. 2024 Jul 30;56:56. doi: 10.1186/s12711-024-00905-7 (PMC11290166; doi:10.1186/s12711-024-00905-7)
Supplement: Supplementary file 14 — Additional file 14: Table S12. KEGG Mapper pathway results for query of Rambouillet-Dorper genes against the Homo sapiens reference database. [file 12711_2024_905_MOESM14_ESM.docx]

| **Rambouillet-Dorper F_ST_ KEGG Mapper Pathway** | **Genes** |
| --- | --- |
| 2-Oxocarboxylic acid metabolism | *GPT* |
| Adrenergic signaling in cardiomyocytes; Arrhythmogenic right ventricular cardiomyopathy; Cardiac muscle contraction; Dilated cardiomyopathy; Hypertrophic cardiomyopathy; Oxytocin signaling pathway | *CACNA1C* |
| Alanine, aspartate and glutamate metabolism; Arginine biosynthesis | *GLUD1, GPT* |
| Alcoholic liver disease | *IL17RA, TRAF3* |
| Alcoholism | *GRIN2B, HDAC7* |
| Aldosterone synthesis and secretion; cGMP-PKG signaling pathway; Cholinergic synapse; Cortisol synthesis and secretion; Cushing syndrome; Insulin secretion; Renin secretion; Serotonergic syneunapse; Taste transduction; Type II diabetes mellitus; Vascular smooth muscle contraction | *CACNA1C* |
| Alzheimer disease | *GRIN2B, NDUFA1, SLC39A4, PSMD3, SNCA, CACNA1C* |
| Amino sugar and nucleotide sugar metabolism; Biosynthesis of nucleotide sugars | *GMDS, UAP1, UGDH* |
| Amphetamine addiction; cAMP signaling pathway; Circadian entrainment; Dopaminergic synapse; Long-term potentiation | *GRIN2B, CACNA1C* |
| AMPK signaling pathway; Galactose metabolism; Glycolysis / Gluconeogenesis; Pentose phosphate pathway; RNA degradation | *PFKM* |
| Amyotrophic lateral sclerosis | *ALYREF, GRIN2B, NDUFA1, PSMD3* |
| Arginine and proline metabolism | *GATM, LAP3, PYCR1* |
| Ascorbate and aldarate metabolism; Pentose and glucuronate interconversions | *UGDH* |
| Autophagy - animal | *BNIP3* |
| Axon guidance | *SLIT2* |
| Biosynthesis of amino acids | *GPT, PFKM, PYCR1* |
| Biosynthesis of cofactors | *LIAS, UGDH* |
| Biosynthesis of unsaturated fatty acids; Fatty acid elongation; Ovarian steroidogenesis | *ACOT4, ACOT1* |
| Breast cancer; Pancreatic cancer | *BRCA2* |
| Calcium signaling pathway | *PDGFRA, STIM2, CACNA1C* |
| Carbon metabolism | *GLUD1, GPT, PFKM* |
| Cell cycle | *MCM4, ANAPC11* |
| Cellular senescence | *RBBP4* |
| Central carbon metabolism in cancer | *PDGFRA, PFKM* |
| Chemical carcinogenesis - reactive oxygen species; Non-alcoholic fatty liver disease | *NDUFA1* |
| Chemical carcinogenesis - receptor activation | *VDR, CACNA1C* |
| Choline metabolism in cancer; Gap junction; Glioma; Human cytomegalovirus infection; Phospholipase D signaling pathway; Prostate cancer; Regulation of actin cytoskeleton | *PDGFRA* |
| Circadian rhythm | *NR1D1* |
| Cocaine addiction; Nicotine addiction | *GRIN2B* |
| Coronavirus disease - COVID-19 | *CSF3, IL6ST, RPL10A, RPL6, RPL8, RPL9, RPS8, TRAF3* |
| Cytokine-cytokine receptor interaction | *IL31RA, CSF3, IL17RA, GHR, IL6ST, BMPR1A, TNFSF18* |
| Diabetic cardiomyopathy | *MPC2, NDUFA1* |
| DNA replication | *MCM4* |
| ECM-receptor interaction | *COL2A1* |
| EGFR tyrosine kinase inhibitor resistance | *NF1, PDGFRA* |
| Endocrine and other factor-regulated calcium reabsorption; Tuberculosis | *VDR* |
| Endocytosis | *CHMP4B, VPS28, PDGFRA, RAB11FIP4, HGS* |
| Epstein-Barr virus infection | *PSMD3, TRAF3* |
| Fanconi anemia pathway | *CENPX, BRCA2* |
| Fluid shear stress and atherosclerosis; Hippo signaling pathway; TGF-beta signaling pathway | *BMPR1A* |
| Focal adhesion | *COL2A1, PDGFRA* |
| FoxO signaling pathway; Shigellosis | *BNIP3* |
| Fructose and mannose metabolism | *GMDS, PFKM* |
| GABAergic synapse | *SLC38A2, CACNA1C, SLC38A1* |
| Glucagon signaling pathway | *GCGR, PFKM* |
| Glutamatergic synapse | *GRIN2B, SLC38A2, CACNA1C, SLC38A1* |
| Glutathione metabolism | *LAP3* |
| Glycerophospholipid metabolism; Phosphonate and phosphinate metabolism | *PCYT2* |
| Glycine, serine and threonine metabolism | *GATM* |
| GnRH secretion | *HCN1, CACNA1C* |
| GnRH signaling pathway | *CACNA1C* |
| Growth hormone synthesis, secretion and action | *GHR, CACNA1C* |
| Hematopoietic cell lineage; Malaria | *CSF3* |
| Hepatitis B; Hepatitis C; Influenza A; Measles; NOD-like receptor signaling pathway; RIG-I-like receptor signaling pathway | *TRAF3* |
| Herpes simplex virus 1 infection | *ALYREF, ZNF641, TRAF3, ZNF7, ZNF34* |
| HIF-1 signaling pathway | *PFKM* |
| Homologous recombination | *RAD54B, BRCA2* |
| Human papillomavirus infection | *COL2A1, TRAF3* |
| Human T-cell leukemia virus 1 infection | *ANAPC11, RAN* |
| Huntington disease | *GRIN2B, NDUFA1, PSMD3* |
| IL-17 signaling pathway | *CSF3, IL17RA, TRAF3* |
| JAK-STAT signaling pathway | *IL31RA, CSF3, GHR, IL6ST, PDGFRA* |
| Kaposi sarcoma-associated herpesvirus infection | *IL6ST, TRAF3* |
| Legionellosis | *EEF1A1, BNIP3* |
| Leishmaniasis | *EEF1A1* |
| Lipid and atherosclerosis | *ERO1A, TRAF3* |
| Lipoic acid metabolism | *LIAS* |
| Long-term depression | *GRID2* |
| MAPK signaling pathway | *NF1, PDGFRA, CACNA1C* |
| Maturity onset diabetes of the young | *PAX6* |
| Melanoma | *PDGFRA* |
| Metabolic pathways | *LIAS, ACOT4, CANT1, COX11, GATM, GLUD1, GMDS, GPT, NDUFA1, LAP3, PDE6G, SIRT7, ADA2, PFKM, PYCR1, PCYT2, ACOT1, UAP1, UGDH* |
| MicroRNAs in cancer | *FOXP1, HNRNPK, PDGFRA* |
| Mineral absorption | *SLC26A3, SLC39A4, VDR* |
| Mitophagy - animal | *BNIP3* |
| mRNA surveillance pathway | *ALYREF, SSU72, CPSF1* |
| mTOR signaling pathway | *SLC38A9* |
| Necroptosis | *CHMP4B, GLUD1, PPIA* |
| Neuroactive ligand-receptor interaction | *NPY4R2, RXFP2, NPB, GCGR, GHR, GRID2, GRIN2B, THRA* |
| Neurotrophin signaling pathway; Vasopressin-regulated water reabsorption | *ARHGDIA* |
| Neutrophil extracellular trap formation | *HDAC7* |
| NF-kappa B signaling pathway; Small cell lung cancer; TNF signaling pathway; Toll-like receptor signaling pathway | *TRAF3* |
| Nicotinate and nicotinamide metabolism | *SIRT7* |
| Nitrogen metabolism | *GLUD1* |
| Notch signaling pathway | *NUMB* |
| Nucleocytoplasmic transport | *ALYREF, EEF1A1, RAN* |
| Nucleotide metabolism | *CANT1, ADA2* |
| Olfactory transduction | *OR52M1, OR2D3, OR52B4, OR10A5, OR2AG1, OR10A2, OR8S1, OR52K1, OR51G2* |
| Oocyte meiosis; Progesterone-mediated oocyte maturation | *ANAPC11* |
| Other glycan degradation | *ENGASE* |
| Oxidative phosphorylation | *COX11, NDUFA1* |
| Pancreatic secretion | *SLC26A3* |
| Parathyroid hormone synthesis, secretion and action | *VDR* |
| Parkinson disease | *NDUFA1, SLC39A4, PSMD3, SNCA* |
| Pathogenic Escherichia coli infection | *TMBIM6* |
| Pathways in cancer | *IL6ST, PDGFRA, BRCA2, TRAF3* |
| Pathways of neurodegeneration - multiple diseases | *GRIN2B, NDUFA1, PSMD3, SNCA, CACNA1C* |
| Phagosome | *HGS* |
| Phototransduction | *PDE6G* |
| PI3K-Akt signaling pathway | *COL2A1, CSF3, GHR, PDGFRA* |
| Prion disease | *GRIN2B, NDUFA1, PSMD3, CACNA1C* |
| Proteasome | *PSMD3* |
| Protein digestion and absorption | *COL2A1, SLC38A2* |
| Protein processing in endoplasmic reticulum | *ERO1A, P4HB, NPLOC4* |
| Proximal tubule bicarbonate reclamation | *SLC25A10, GLUD1* |
| Purine metabolism | *CANT1, PDE6G, ADA2* |
| Pyrimidine metabolism | *CANT1* |
| Rap1 signaling pathway | *GRIN2B, EVL, PDGFRA* |
| Ras signaling pathway | *GRIN2B, NF1, PDGFRA* |
| Relaxin signaling pathway | *RXFP2* |
| Retrograde endocannabinoid signaling | *NDUFA1, CACNA1C* |
| Ribosome | *RPL10A, MRPS18C, RPL6, RPL8, RPL9, MRPL12, RPS8* |
| Ribosome biogenesis in eukaryotes | *RAN* |
| Signaling pathways regulating pluripotency of stem cells | *IL6ST, PAX6, BMPR1A* |
| Spinocerebellar ataxia | *GRIN2B, PSMD3* |
| Spliceosome | *ALYREF, PRPF40B, HNRNPK* |
| Systemic lupus erythematosus | *GRIN2B, TRIM21* |
| Th17 cell differentiation; Viral protein interaction with cytokine and cytokine receptor | *IL6ST* |
| Thermogenesis | *COX11, KLB, NDUFA1* |
| Thyroid hormone signaling pathway | *PFKM, THRA, MED24* |
| Thyroid hormone synthesis | *DUOXA2, DUOX2, SLC26A4, DUOX1* |
| Tight junction | *EPB41L4B* |
| Transcriptional misregulation in cancer | *ASPSCR1* |
| Ubiquitin mediated proteolysis | *UBE2E3, UBE2K, ANAPC11* |
| Vibrio cholerae infection | *ERO1A* |
| Viral carcinogenesis | *HNRNPK, IL6ST, HDAC7, TRAF3* |
| Viral life cycle - HIV-1 | *PSIP1, CHMP4B, PPIA, RAN, HGS* |
| Wnt signaling pathway | *NOTUM* |
