# Supplementary material for: Genetic diversity of United States Rambouillet, Katahdin and Dorper sheep
Source: Genet Sel Evol. 2024 Jul 30;56:56. doi: 10.1186/s12711-024-00905-7 (PMC11290166; doi:10.1186/s12711-024-00905-7)
Supplement: Supplementary file 15 — Additional file 15: Table S13. Most significantly enriched GO biological process function terms from Dorper ROH islands. Gene names were searched against the Bos taurus reference database. Each term in italics represents the most specific subclass with related parent terms directly below. [file 12711_2024_905_MOESM15_ESM.docx]

| **GO: Biological Process** | **Ref #** | **Query #** | **Expected** | **Fold Enrichment** | **+/-** | **FDR** |
| --- | --- | --- | --- | --- | --- | --- |
| *Keratinization* | 55 | 25 | 1.17 | 21.29 | + | 1.02E-18 |
| Keratinocyte differentiation | 104 | 26 | 2.22 | 11.71 | + | 6.76E-15 |
| Epidermal cell differentiation | 157 | 26 | 3.35 | 7.76 | + | 3.33E-11 |
| Epithelial cell differentiation | 490 | 33 | 10.46 | 3.15 | + | 2.63E-05 |
| Epithelium development | 878 | 46 | 18.74 | 2.45 | + | 6.56E-05 |
| Tissue development | 1395 | 59 | 29.78 | 1.98 | + | 7.85E-04 |
| Epidermis development | 236 | 29 | 5.04 | 5.76 | + | 7.18E-10 |
| Skin development | 217 | 29 | 4.63 | 6.26 | + | 1.21E-10 |
| *Intermediate filament organization* | 84 | 26 | 1.79 | 14.5 | + | 9.49E-17 |
| Intermediate filament cytoskeleton organization | 101 | 29 | 2.16 | 13.45 | + | 6.97E-18 |
| Intermediate filament-based process | 102 | 29 | 2.18 | 13.32 | + | 5.86E-18 |
| Cytoskeleton organization | 1207 | 67 | 25.77 | 2.6 | + | 7.15E-09 |
| Organelle organization | 2914 | 114 | 62.21 | 1.83 | + | 3.35E-07 |
| Cellular component organization | 5179 | 161 | 110.57 | 1.46 | + | 2.82E-04 |
| Cellular component organization or biogenesis | 5426 | 169 | 115.84 | 1.46 | + | 1.11E-04 |
| Supramolecular fiber organization | 543 | 41 | 11.59 | 3.54 | + | 2.24E-08 |
| *Detection of chemical stimulus involved in sensory perception of smell* | 1045 | 5 | 22.31 | 0.22 | - | 8.33E-03 |
| Detection of chemical stimulus involved in sensory perception | 1086 | 5 | 23.18 | 0.22 | - | 5.12E-03 |
| Detection of stimulus involved in sensory perception | 1135 | 5 | 24.23 | 0.21 | - | 2.23E-03 |
| Detection of stimulus | 1221 | 7 | 26.07 | 0.27 | - | 6.15E-03 |
| Detection of chemical stimulus | 1111 | 5 | 23.72 | 0.21 | - | 3.88E-03 |
