# Supplementary material for: Genetic diversity of United States Rambouillet, Katahdin and Dorper sheep
Source: Genet Sel Evol. 2024 Jul 30;56:56. doi: 10.1186/s12711-024-00905-7 (PMC11290166; doi:10.1186/s12711-024-00905-7)
Supplement: Supplementary file 16 — Additional file 16: Table S14. Significantly enriched GO cellular component terms from Dorper ROH islands. Gene names were searched against the Bos taurus reference database. Each term in italics represents the most specific subclass with related parent terms directly below. [file 12711_2024_905_MOESM16_ESM.docx]

| **GO: Cellular Component** | ***Bos taurus* Ref #** | **Query #** | **Expected** | **Fold Enrichment** | **+/-** | **FDR** |
| --- | --- | --- | --- | --- | --- | --- |
| *Keratin filament* | 99 | 28 | 2.11 | 13.25 | + | 1.30E-17 |
| Intermediate filament | 166 | 29 | 3.54 | 8.18 | + | 1.09E-13 |
| Polymeric cytoskeletal fiber | 681 | 44 | 14.54 | 3.03 | + | 7.24E-08 |
| Cytoskeleton | 2143 | 91 | 45.75 | 1.99 | + | 1.05E-07 |
| Intracellular non-membrane-bounded organelle | 4563 | 168 | 97.41 | 1.72 | + | 8.67E-11 |
| Intracellular organelle | 12,977 | 349 | 277.04 | 1.26 | + | 3.55E-08 |
| Intracellular anatomical structure | 14,893 | 398 | 317.95 | 1.25 | + | 2.47E-11 |
| Cellular anatomical entity | 20,614 | 480 | 440.09 | 1.09 | + | 2.17E-06 |
| Organelle | 13,326 | 353 | 284.5 | 1.24 | + | 1.68E-07 |
| Non-membrane-bounded organelle | 4564 | 168 | 97.44 | 1.72 | + | 7.30E-11 |
| Supramolecular fiber | 873 | 48 | 18.64 | 2.58 | + | 1.35E-06 |
| Supramolecular polymer | 883 | 48 | 18.85 | 2.55 | + | 1.57E-06 |
| Supramolecular complex | 1207 | 57 | 25.77 | 2.21 | + | 5.31E-06 |
| Intermediate filament cytoskeleton | 195 | 30 | 4.16 | 7.21 | + | 4.62E-13 |
| *Cornified envelope* | 29 | 5 | 0.62 | 8.08 | + | 3.77E-02 |
| Cytosolic large ribosomal subunit | 136 | 13 | 2.9 | 4.48 | + | 1.19E-03 |
| Cytosolic ribosome | 237 | 22 | 5.06 | 4.35 | + | 3.76E-06 |
| Ribosome | 414 | 26 | 8.84 | 2.94 | + | 2.21E-04 |
| Cytosol | 3500 | 115 | 74.72 | 1.54 | + | 2.45E-04 |
| Cytoplasm | 11,307 | 300 | 241.39 | 1.24 | + | 2.86E-05 |
| Large ribosomal subunit | 210 | 16 | 4.48 | 3.57 | + | 1.69E-03 |
| Ribosomal subunit | 351 | 26 | 7.49 | 3.47 | + | 1.32E-05 |
| Ribonucleoprotein complex | 980 | 40 | 20.92 | 1.91 | + | 1.14E-02 |
| Protein-containing complex | 5844 | 166 | 124.76 | 1.33 | + | 3.14E-03 |
| *Cytosolic small ribosomal subunit* | 101 | 9 | 2.16 | 4.17 | + | 3.01E-02 |
| Nucleoplasm | 3207 | 98 | 68.47 | 1.43 | + | 1.86E-02 |
| Nuclear lumen | 3670 | 115 | 78.35 | 1.47 | + | 1.75E-03 |
| Intracellular organelle lumen | 4153 | 131 | 88.66 | 1.48 | + | 3.25E-04 |
| Organelle lumen | 4153 | 131 | 88.66 | 1.48 | + | 3.40E-04 |
| Membrane-enclosed lumen | 4153 | 131 | 88.66 | 1.48 | + | 3.11E-04 |
| Nucleus | 7024 | 194 | 149.95 | 1.29 | + | 2.44E-03 |
