# Supplementary material for: Genetic diversity of United States Rambouillet, Katahdin and Dorper sheep
Source: Genet Sel Evol. 2024 Jul 30;56:56. doi: 10.1186/s12711-024-00905-7 (PMC11290166; doi:10.1186/s12711-024-00905-7)
Supplement: Supplementary file 17 — Additional file 17: Table S15. Significantly enriched GO cellular molecular function terms from Dorper ROH islands. Gene names were searched against the Bos taurus reference database. Each term in italics represents the most specific subclass with related parent terms directly below. [file 12711_2024_905_MOESM17_ESM.docx]

| **GO: Molecular Function** | **Ref #** | **Query #** | **Expected** | **Fold Enrichment** | **+/-** | **FDR** |
| --- | --- | --- | --- | --- | --- | --- |
| *Structural constituent of skin epidermis* | 40 | 25 | 0.85 | 29.28 | + | 7.73E-22 |
| Structural molecule activity | 905 | 61 | 19.32 | 3.16 | + | 3.32E-11 |
| *Integrin binding* | 130 | 12 | 2.78 | 4.32 | + | 4.16E-02 |
| *Structural constituent of ribosome* | 359 | 24 | 7.66 | 3.13 | + | 3.19E-03 |
| *G protein-coupled receptor activity* | 1506 | 12 | 32.15 | 0.37 | - | 4.51E-02 |
| *Olfactory receptor activity* | 1043 | 5 | 22.27 | 0.22 | - | 2.35E-02 |
