# Supplementary material for: Genetic diversity of United States Rambouillet, Katahdin and Dorper sheep
Source: Genet Sel Evol. 2024 Jul 30;56:56. doi: 10.1186/s12711-024-00905-7 (PMC11290166; doi:10.1186/s12711-024-00905-7)
Supplement: Supplementary file 18 — Additional file 18: Table S16. KEGG Mapper pathway enrichment for genes located within Dorper ROH islands. KEGG query was made against the Homo sapiens reference. [file 12711_2024_905_MOESM18_ESM.docx]

| **Dorper ROH KEGG Mapper Pathway**  **(Gene #)** | **Pathway Genes** |
| --- | --- |
| Metabolic pathways (38) | *PLA2G4B, ATP5MG, ADCY6, GALNT6, PLA2G4E, COX7A2, DNMT1, RPIA, PLA2G4F, MGAT4C, GANC, GLUD1, PLA2G4D, ITPKA, LALBA, MDH1, ATP12A, OAT, RDH8, COQ6, LAP3, CRYL1, CSAD, PDE4A, PDE1B, ATP5MC2, PFKM, CYCS, NDUFA12, URAD, TPI1, PRDM2, CHAC1, KMT2D, GALNT4, CERS5, MTMR6, KL* |
| Coronavirus disease (19) | *NLRP3, RPL10A, RPL10, RPL15, RPL17, RPL19, RPL21, RPL27, RPL32, RPL37, RPL36A, RPS2, RPS3A, RPS6, RPS11, RPS23, RPS24, C3, TYK2* |
| Ribosome (17) | *RPL10A, MRPL4, RPL10, RPL15, RPL17, RPL19, RPL21, RPL27, RPL32, RPL37, RPL36A, RPS2, RPS3A, RPS6, RPS11, RPS23, RPS24* |
| Pathways of neurodegeneration (15) | *TUBA1B, COX7A2, SLC25A5, ATP5MC2, CYCS, NDUFA12, PRPH, PSMB6, RAF1, ATG101, WNT1, WNT7A, WNT10B, TUBA1A, TUBA1C* |
| Alzheimer disease (15) | *TUBA1B, COX7A2, SLC25A5, SLC11A2, ATP5MC2, CYCS, NDUFA12, PSMB6, RAF1, ATG101, WNT1, WNT7A, WNT10B, TUBA1A, TUBA1C* |
| PI3K-Akt signaling pathway (14) | *CDC37, COL2A1, EIF4B, FGF9, FLT1, FLT3, NR4A1, IBSP, ITGA5, ITGB7, KITLG, RAF1, RPS6, SPP1* |
| Pathways in cancer (14) | *ADCY6, FGF9, FLT3, KITLG, CYCS, DLL4, PPARG, RAF1, SP1, BRCA2, TPM3, WNT1, WNT7A, WNT10B* |
| MAPK signaling pathway (13) | *PLA2G4B, PLA2G4E, DUSP6, FGF9, FLT1, FLT3, PLA2G4F, PLA2G4D, NR4A1, KITLG, RAF1, MAP3K12, CACNB3* |
| Huntington disease (13) | *TUBA1B, COX7A2, SLC25A5, ATP5MC2, CYCS, PPARG, NDUFA12, PSMB6, ATG101, SP1, TAF4, TUBA1A, TUBA1C* |
| Amyotrophic lateral sclerosis (12) | *TUBA1B, COX7A2, HNRNPA1, ATP5MC2, CYCS, NDUFA12, PRPH, PSMB6, ATG101, TUBA1A, TUBA1C, NUP58* |
| Thermogenesis (11) | *ATP5MG, ADCY6, COX7A2, NDUFAF1, ATP5MC2, PPARG, PRKAG1, NDUFA12, RPS6, SMARCD1, COX14* |
| Parkinson disease (10) | *TUBA1B, COX7A2, SLC25A5, SLC11A2, ATP5MC2, CYCS, NDUFA12, PSMB6, TUBA1A, TUBA1C* |
| Salmonella infection (10) | *ARPC2, TUBA1B, WASF3, NLRP3, VPS39, NCKAP1L, CYCS, RAF1, TUBA1A, TUBA1C* |
| Human papillomavirus infection (10) | *COL2A1, IBSP, ITGA5, ITGB7, RAF1, SPP1, TYK2, WNT1, WNT7A, WNT10B* |
| Necroptosis (10) | *PLA2G4B, NLRP3, PLA2G4E, FTH1, PLA2G4F, GLUD1, PLA2G4D, SLC25A5, HMGB1, TYK2* |
| Herpes simplex virus 1 infection (10) | *ZNF891, ZNF641, ZNF846, ITGA5, ZNF699, CYCS, ZNF317, C3, TYK2, ZNF85* |
| Olfactory transduction (10) | *OR10AD1, OR7D4, OR7G1, OR7D2, OR7E24, OR7C2, OR8S1, OR7G2, OR7G3, PDE1B* |
